# Partially Ordered Lanthanide Carboxylates with a Highly Adaptable 1D Polymeric Structure

**DOI:** 10.3390/polym14163328

**Published:** 2022-08-16

**Authors:** Dimitry Grebenyuk, Mirijam Zobel, Dmitry Tsymbarenko

**Affiliations:** 1 Department of Chemistry, Lomonosov Moscow State University, Leninskie gory 1, 119991 Moscow, Russia; 2 Institute of Crystallography, RWTH Aachen University, Jägerstr. 17-19, 52066 Aachen, Germany

**Keywords:** coordination polymers, structure elucidation, rare earths, luminescence, synthetic methods

## Abstract

A new family of 14 isostructural [Ln(piv)_3_(en)]_∞_ lanthanide pivalate (piv^−^, 2,2-dimethylpropanoate) complexes with ethylenediamine (en) was synthesized by a topology-preserving transformation from 1D coordination polymers [Ln(piv)_3_]_∞_. The crystal structures of the compounds were determined by single-crystal and powder X-ray diffraction, which demonstrated that despite the regular ligand arrangement within the chains, the latter are intricately packed within the partially ordered crystal, as only two of four ligands are strictly bound by the translational symmetry. The peculiarities of the lanthanide coordination environment were explored by total X-ray scattering with pair distribution function analysis. Periodic DFT calculations revealed the chain stabilization by intrachain H-bonds and weak interchain interactions. Noticeably, the energy difference was infinitesimally small even between the two considered extreme variants of ordered packing, which is in line with the disturbed packing order of the chains. The luminescent properties of Eu and Tb complexes were investigated in order to prove the energy transfer between lanthanide ions within the heterometallic complex. This opens up the prospect of creating new materials for optical applications. The heterometallic compound Eu_0.05_Tb_0.95_(piv)_3_(en) was synthesized, and was found to demonstrate temperature-dependent luminescence with a linear dependence of the thermometric parameter I(Eu)/I(Tb) within the temperature range from −80 °C to 80 °C, and had a maximum relative sensitivity value of 0.2%/K.

## 1. Introduction

Coordination polymers attract a great deal of attention due to their numerous potential applications as catalysts [1,2,3,4], in gas sorption and separation [5,6,7,8], or as luminescent and sensing materials [9,10,11,12,13,14]. The coordination polymers of lanthanides are of particular interest because of the exciting magnetic and luminescent features of lanthanide ions. At the same time, high coordination numbers of lanthanides, their ionic bonding nature and, subsequently, their structural flexibility complicate the design and deliberate synthesis of such compounds.

The luminescent and magnetic features of lanthanide-based materials crucially depend on the central ion, as well as on the ligand environment. The properties of the synthesized compounds could be altered by the fine tuning of the coordination sphere, e.g., the introduction of a neutral ligand. However, neutral ligand molecules often preferentially saturate the coordination sphere of the lanthanide replacing initial bridging ligands in the coordination polymers. This results in the destruction of the polymeric architecture and leads to complexes with lower dimensionality (down to 0D). Therefore, the processes which can introduce the ligand into the polymer without the cleavage of the coordination polymer could be promising tools for the design of new lanthanide compounds. Applications of the materials with optical response (luminescent thermometry, luminescent sensing, up-conversion, etc.) based on lanthanide coordination compounds usually require the uniform distribution and the proximity of several distinct lanthanides—which are often significantly different in their ionic radii—within the same substance. This poses a challenge for the design of new isostructural lanthanide platforms, with only a few reported examples so far [15,16], as the crystal structure of the lanthanide complexes tends to change along the lanthanide series with the change of the ionic radius.

One of the promising applications of mixed-metal lanthanide complexes is their use in double-ion ratiometric luminescent thermometers. The development strategy of ratiometric metal-based luminescent thermometers is driven by the possibility of temperature-dependent energy transfer between luminescent centers within the crystal structure. The efficiency of the transfer crucially depends on several factors, such as the distance between luminescent centers, the relative positions of the corresponding energy levels, or the pathways for luminescence quenching. The Eu/Tb pair has been extensively explored as a platform for luminescent thermometry [17,18,19,20] due to the temperature-sensitive energy transfer between the ^5^D_0_ level of Eu^3+^ (17,300 cm^−1^) and the ^5^D_4_ level of Tb^3+^ (20,500 cm^−1^).

In this paper, we wish to report a new family of isostructural lanthanide pivalate mixed-ligand 1D coordination polymers with ethylenediamine synthesized for the whole lanthanide series by a simple topology-preserving transformation. The luminescent properties of Eu and Tb complexes are examined, and the heterometallic compound Eu_0.05_Tb_0.95_(piv)_3_(en) is proposed as a potential luminescent thermometer.

## 2. Materials and Methods

The rare earth nitrates (Reakhim, Moscow, Russia), pivalic acid (Merck, Darmstadt Germany, 98%), ethylenediamine (Sigma-Aldrich, Darmstadt, Germany, 99%), ethylenediamine (Sigma-Aldrich, Darmstadt, Germany, 99%), acetonitrile (Komponent-Reaktiv, Moscow, Russia, 99.85%), and benzene (Komponent-Reaktiv, Moscow, Russia, 99.6%)—all of analytical grade—were used without further purification. The Ln(piv)_3_ powders were prepared and characterized as described earlier [21].

The Ln content was measured by ICP-MS on a quadruple ICP-MS spectrometer Agilent 7500c (Agilent Technologies, Tokyo, Japan). The following isotopes were considered: ^141^Pr, ^151^Eu, ^153^Eu, and ^159^Tb. ICP-MS-68A (High-Purity Standards, Charleston, USA) containing 10 mg L^−1^ of each element was used for the preparation of the calibration solutions. The composition of the compounds was determined by CHN and thermogravimetric analyses. The TG-DTA data were collected in the atmosphere of air using a Derivatograph Q-1500 D (heating rate, 10°/min; mass of sample, 50 mg). CHN analysis was carried out on an Elementar Vario Micro Cube. The FT-IR spectra were recorded on a Perkin-Elmer Spectrum One FTIR spectrometer and on a Shimadzu IRAffinity-1S spectrometer in the attenuated total reflectance (ATR) geometry in the 650–4000 cm^−1^ range of wave numbers.

Phase identification was performed by means of powder XRD data. The powder XRD data were collected at room temperature on a Rigaku SmartLab diffractometer (9 kW rotating anode, CuKα radiation, secondary graphite monochromator) in reflection θ-θ geometry, and on a STOE Stadi P diffractometer (curved Ge monochromator, CuKα_1_, λ = 1.5406 Å) in transmission geometry.

**Synthesis**

First, [Ln(piv)_3_] (0.53 mmol) was dispersed in 11 mL of a 10:1 vol. mixture of CH_3_CN and C_6_H_6_. Ethylenediamine (36 μL, 0.53 mmol) was added, and the dispersion was stirred for 2 h at 80 °C. The crystalline precipitate was filtered off, dried, and stored in air. The yield was ca. 90%.

**La(piv)_3_(en)**

PXRD (300 K): Space group *Iba*2, a = 12.672 (5) Å, b = 17.750 (12) Å, c = 10.274 (10) Å. Thermogravimetric analysis (weight residue, %) was calculated for the decomposition of LaC_17_H_35_N_2_O_6_ (502.38) to 1/2 La_2_O_3_ (162.90) 0.324; we found 0.343. FT-IR (ATR, ν, cm^−1^): 3364w, 3341w, 3273w, 2954m, 2915m, 2870m (νNH, νCH); 1516s (ν_as_COO), 1481s, 1417s (ν_s_COO), 1374s, 1354s, 1223s (νCN, νCC), 1132w, 1011s, 977s, 946s, 888s, 849w, and 789s. 

**Ce(piv)_3_(en)**

PXRD (300 K): Space group *Iba*2, a = 12.664 (3) Å, b = 17.856(5) Å, c = 10.258(3) Å. Thermogravimetric analysis (weight residue, %) was calculated for the decomposition of CeC_17_H_35_N_2_O_6_ (503.59) to CeO_2_ (172.11) 0.342; we found 0.337. FT-IR (ATR, ν, cm^−1^): 3366w, 3343w, 3269w, 2955m, 2915m, 2871m (νNH, νCH); 2375w, 2349w, 2314w, 1519s (ν_as_COO), 1482s, 1416s (ν_s_COO), 1373s, 1355s, 1224s (νCN, νCC), 1010s, 978s, 946s, 888s, 803s, and 788s. 

**Pr(piv)_3_(en)**

PXRD (300 K): Space group *Iba*2, a = 12.5739 (18) Å, b = 17.785(3) Å, c = 10.280(2) Å. Thermogravimetric analysis (weight residue, %) was calculated for the decomposition of PrC_17_H_35_N_2_O_6_ (504.38) to 1/6 Pr_6_O_11_ (170.24) 0.337; we found 0.342. FT-IR (ATR, ν, cm^−1^): 3364w, 3340w, 3268w, 3168w, 2953m, 2915m, 2868m (νNH, νCH); 2374w, 2348w, 2320w, 2306w, 1519s (ν_as_COO), 1482s, 1416s (ν_s_COO), 1371s, 1354s, 1224s (νCN, νCC), 1133w, 1010s, 978s, 945s, 888s, 804s, and 788s. 

**Nd(piv)_3_(en)**

PXRD (300 K): Space group *Iba*2, a = 12.543 (3) Å, b = 17.812(5) Å, c = 10.235(5) Å. Thermogravimetric analysis (weight residue, %) was calculated for the decomposition of NdC_17_H_35_N_2_O_6_ (507.71) to 1/2 Nd_2_O_3_ (168.24) 0.331; we found 0.348. FT-IR (ATR, ν, cm^−1^): 3363w, 3340w, 3263w, 2953m, 2914m, 2868m (νNH, νCH); 2374w, 2349w, 2320w, 2309w, 1523s (ν_as_COO), 1479s, 1418s (ν_s_COO), 1371s, 1354s, 1224s (νCN, νCC), 1012s, 982s, 947s, 889s, 803s, and 788s. 

**Sm(piv)_3_(en)**

PXRD (300 K): Space group *Iba*2, a = 12.511 (5) Å, b = 17.860(10) Å, c = 10.200(4) Å. Thermogravimetric analysis (weight residue, %) was calculated for the decomposition of SmC_17_H_35_N_2_O_6_ (513.84) to 1/2 Sm_2_O_3_ (174.37) 0.339; we found 0.337. FT-IR (ATR, ν, cm^−1^): 3362w, 3341w, 3263w, 3167w, 2951m, 2918m, 2869m (νNH, νCH); 2375w, 2350w, 2320w, 1547s, 1525s (ν_as_COO), 1481s, 1457m, 1419s (ν_s_COO), 1372s, 1354s, 1224s (νCN, νCC), 1014s, 984s, 947s, 890s, 804s, 788s. 

**Eu(piv)_3_(en)**

PXRD (300 K): Space group *Iba*2, a = 12.5054 (16) Å, b = 17.872(2) Å, c = 10.2111(15) Å. Thermogravimetric analysis (weight residue, %) was calculated for the decomposition of EuC_17_H_35_N_2_O_6_ (515.43) to 1/2 Eu_2_O_3_ (175.96) 0.341; we found 0.348. FT-IR (ATR, ν, cm^−1^): 3365w, 3342w, 3263w, 3167w, 2955m, 2917m, 2871m (νNH, νCH); 2376w, 2350w, 2306w, 1549s, 1525s(ν_as_COO), 1482s, 1455m, 1420s (ν_s_COO), 1371s, 1356s, 1225s (νCN, νCC), 1014s, 984s, 948s, 890s, 804s, and 787s. 

**Gd(piv)_3_(en)**

PXRD (300 K): Space group *Iba*2, a = 12.456 (4) Å, b = 17.819(8) Å, c = 10.205(4) Å. Thermogravimetric analysis (weight residue, %) was calculated for the decomposition of GdC_17_H_35_N_2_O_6_ (520.72) to 1/2 Gd_2_O_3_ (181.25) 0.348; we found 0.344. FT-IR (ATR, ν, cm^−1^): 3366w, 3345w, 3265w, 3169w, 2955m, 2921m, 2873m (νNH, νCH); 1555s, 1529s (ν_as_COO), 1482s, 1457m, 1421s (ν_s_COO), 1374s, 1356s, 1228s (νCN, νCC), 1016s, 988s, 950s, 892s, 806s, and 789s. 

**Tb(piv)_3_(en)**

PXRD (300 K): Space group *Iba*2, a = 12.4359(15) Å, b = 17.825(2) Å, c = 10.1920(13) Å. Thermogravimetric analysis (weight residue, %) was calculated for the decomposition of TbC_17_H_35_N_2_O_6_ (522.40) to 1/4Tb_4_O_7_ (186.92) 0.358; we found 0.365. FT-IR (ATR, ν, cm^−1^): 3364w, 3342w, 3265w, 3169w, 2955m, 2921m, 2872m (νNH, νCH); 1553s, 1527s (ν_as_COO), 1481s, 1456s, 1421s (ν_s_COO), 1372s, 1355s, 1226s (νCN, νCC), 1016s, 988s, 950s, 891s, 805s, and 788s. 

**Dy(piv)_3_(en)**

PXRD (300 K): Space group *Iba*2, a = 12.416 (5) Å, b = 17.804(8) Å, c = 10.158(4) Å. Thermogravimetric analysis (weight residue, %) was calculated for the decomposition of DyC_17_H_35_N_2_O_6_ (525.97) to 1/2 Dy_2_O_3_ (186.50) 0.354; we found 0.350. FT-IR (ATR, ν, cm^−1^): 3365w, 3343w, 3264w, 3170w, 2956m, 2918m, 2869m (νNH, νCH); 2375w, 2349w, 2319w, 1555s, 1529s (ν_as_COO), 1482s (ν_s_COO), 1457m, 1422s, 1372s, 1355s, 1226s (νCN, νCC), 1018s, 990s, 951s, 936m, 893s, 806s, and 788s.

**Ho(piv)_3_(en)**

PXRD (300 K): Space group *Iba*2, a = 12.406 (8) Å, b = 17.760(13) Å, c = 10.158(8) Å. Thermogravimetric analysis (weight residue, %) was calculated for the decomposition of HoC_17_H_35_N_2_O_6_ (528.40) to 1/2 Ho_2_O_3_ (188.93) 0.358; we found 0.363. FT-IR (ATR, ν, cm^−1^): 3366w, 3346w, 3266w, 2952m, 2920m, 2870w (νNH, νCH); 2373w, 2348w, 2319w, 2309w, 1556s, 1531s (ν_as_COO), 1482s (ν_s_COO), 1456m, 1423s, 1373s, 1355s, 1228s (νCN, νCC), 1018s, 992s, 954s, 935w, 892s, 806s, 792s, and 788s. 

**Er(piv)_3_(en)**

PXRD (300 K): Space group *Iba*2, a = 12.391(2) Å, b = 17.830(3) Å, c = 10.141(3) Å. Thermogravimetric analysis (weight residue, %) was calculated for the decomposition of ErC_17_H_35_N_2_O_6_ (530.73) to 1/2Er_2_O_3_ (191.26) 0.360; we found 0.379. FT-IR (ATR, ν, cm^−1^): 3365w, 3343w, 3264w, 3170w, 2954m, 2918m, 2870m (νNH, νCH); 2373w, 2347w, 2318w, 2309w, 1557s, 1531s (ν_as_COO), 1482s (ν_s_COO), 1456m, 1422s, 1373s, 1355s, 1227s (νCN, νCC), 1135w, 1019s, 992s, 953s, 935m, 893s, 807s, and 788s. 

**Tm(piv)_3_(en)**

PXRD (300 K): Space group *Iba*2, a = 12.3746(3) Å, b = 17.7891(4) Å, c = 10.1367(3) Å. Thermogravimetric analysis (weight residue, %) was calculated for the decomposition of TmC_17_H_35_N_2_O_6_ (532.40) to 1/2Tm_2_O_3_ (192.93) 0.362; we found 0.365. FT-IR (ATR, ν, cm^−1^): 3367w, 3347w, 2952m, 2920m, 2873m (νNH, νCH); 2374w, 2346w, 2319w, 2310w, 1557s, 1532s (ν_as_COO), 1481s (ν_s_COO), 1456m, 1422s, 1373s, 1354s, 1227s (νCN, νCC), 1135w, 1019s, 991s, 954s, 935m, 893s, 807s, 793s, and 788s. 

**Yb(piv)_3_(en)**

PXRD (300 K): Space group *Iba*2, a = 12.3552(20) Å, b = 17.781(2) Å, c = 10.139(5) Å. Thermogravimetric analysis (weight residue, %) was calculated for the decomposition of YbC_17_H_35_N_2_O_6_ (536.52) to 1/2Yb_2_O_3_ (197.05) 0.367; we found 0.370. FT-IR (ATR, ν, cm^−1^): FT-IR (ATR, ν, cm^−1^): 3367w, 3344w, 3266w, 2952m, 2920m, 2872m (νNH, νCH); 2374w, 2346w, 2319w, 2310w, 1558s, 1533s (ν_as_COO), 1506m, 1481s (ν_s_COO), 1456m, 1423s, 1371s, 1354s, 1226s (νCN, νCC), 1135w, 1020s, 993s, 955s, 936m, 894s, 807s, 793s, and 787s. 

**Lu(piv)_3_(en)**

PXRD (300 K): Space group *Iba*2, a = 12.3443(2) Å, b = 17.7621(4) Å, c = 10.1222(5) Å. Thermogravimetric analysis (weight residue, %) was calculated for the decomposition of LuC_17_H_35_N_2_O_6_ (538.44) to 1/2Lu_2_O_3_ (198.97) 0.369; we found 0.365. FT-IR (ATR, ν, cm^−1^): 3368w, 3346w, 3268w, 2953m, 2919m, 2872m (νNH, νCH); 2376w, 2348w, 2319w, 2309w, 1558s, 1532s (ν_as_COO), 1506m, 1482s (ν_s_COO), 1456m, 1424s, 1371s, 1354s, 1228s (νCN, νCC), 1135w, 1020s, 994s, 956s, 935m, 895s, 808s, 794s, and 788s. 

**Eu_0.53_Tb_0.47_(piv)_3_(en)**

PXRD (300 K): Space group *Iba*2, a = 12.477(2) Å, b = 17.815(3) Å, c = 10.210(4) Å. ICP-MS (at% of total metal content): found 0.53 Eu, 0.47 Tb. Elemental analysis (weight %): was calculated for Eu_0.53_Tb_0.47_C_17_H_35_N_2_O_6_ (518.71) 39.36 C, 6.80 H, 5.40 N; found 39.67 C, 6.75 H, 5.62 N. FT-IR (ATR, ν, cm^−1^): 3366w, 3345w, 3268w, 3169w, 2956m, 2920m, 2873m (νNH, νCH); 1550s, 1528s (ν_as_COO), 1481s (ν_s_COO), 1457m, 1420s, 1375s, 1356s, 1226s (νCN, νCC), 1132w, 1015s, 985s, 950s, 891s, 806s, and 789s.

**Eu_0.05_Tb_0.95_(piv)_3_(en)**

PXRD (300 K): Space group *Iba*2, a = 12.4420(13) Å, b = 17.830(2) Å, c = 10.1932(12) Å. ICP-MS (at% of total metal content): found 0.05 Eu, 0.95 Tb. FT-IR (ATR, ν, cm^−1^): 3365w, 3344w, 3267w, 2954m, 2919m, 2870m (νNH, νCH); 2372w, 2346w, 2319w, 2310w, 1553s, 1527s (ν_as_COO), 1481s (ν_s_COO), 1455m, 1421s, 1371s, 1354s, 1226s (νCN, νCC), 1131w, 1016s, 988s, 951s, 935m, 891s, 806s, and 788s.

**Eu_0.01_Tb_0.99_(piv)_3_(en)**

PXRD (300 K): Space group *Iba*2, a = 12.440 (2) Å, b = 17.794(4) Å, c = 10.190(2) Å. ICP-MS (at% of total metal content): found 0.01 Eu, 0.99 Tb. FT-IR (ATR, ν, cm^−1^): 3366w, 3345w, 3266w, 2953m, 2918m, 2869m (νNH, νCH); 2374w, 2348w, 2319w, 2310w, 1553s, 1527s (ν_as_COO), 1481s (ν_s_COO), 1456m, 1421s, 1373s, 1354s, 1227s (νCN, νCC), 1132w, 1016s, 988s, 950s, 935m, 892s, 806s, and 788s.

**Crystallographic Information**

Single crystals of [Eu_0.53_Tb_0.47_(piv)_3_(en)] for X-ray diffraction study were obtained through a reaction between an acetonitrile solution of Ln_6_(OH)_8_(piv)_10_(deta)_4_ (deta = diethylenetriamine) [22] and ethylenediamine generated in situ from deta due to its decomposition. After several hours of heating and subsequent slow cooling, a few tiny needle-like crystals were harvested. Single-crystal X-ray diffraction data were collected on a Bruker SMART APEX II diffractometer with a CCD area detector using Mo Kα radiation (λ = 0.71073 Å, graphite monochromator) in three ω-scans (Δω = 0.5°, 35 s exposition per frame). The crystal was found to be a two-component inversion twin with the volume fraction of components being equal to 0.56(14):0.44(14). The correctness of the selected unit cell was independently confirmed by the ab initio indexing of the PXRD pattern. The diffraction data were corrected for absorption by SADABS. The crystal structure was solved by direct methods and refined anisotropically for all of the non-H atoms using the full-matrix F^2^ least-squares technique (SHELXTL PLUS) [23]. All of the H atoms were placed in geometrically calculated positions, and were refined in a riding mode. Soft constrains were applied for C-C and C-O bond distances, and the O-C-O and C-C-C valence angles of pivalate-anions. 

Powder XRD data for the Rietveld refinement of the [Tm(piv)_3_(en)] and [Lu(piv)_3_(en)] structures were collected on a Rigaku SmartLab diffractometer (9 kW rotating anode, CuKα radiation, secondary graphite monochromator) operated in symmetrical reflection θ-θ mode. The powder sample was placed into a side-loaded sample holder, which was rotating along the φ-axis during the measurements in order to avoid texture and to improve the statistics. The structures were solved using the initial model extracted from the [Eu_0.53_Tb_0.47_(piv)_3_(en)] analog and refined by the full-profile Rietveld method in JANA2006 [24] with soft constrains on the bond distances and valence angles in the piv and en ligands. The Ln atoms were refined anisotropically, while the C and O atoms were refined in isotropic approximation with identical ADP for a certain atomic type; the H atoms were placed in idealized positions and refined in a riding model. The patterns were fitted with an eleven-term Legendre polynomial background and a five-term pseudo-Voigt shape function with asymmetry corrected by axial divergence. The details of the data collection and refinement parameters are summarized in Table 1. Figures S5 and S6 show the refined powder XRD profiles. CCDC 2057567-2057569 contains crystallographic information associated with this paper. 

**Periodic DFT calculations**

DFT calculations of [Ln(piv)_3_(en)] crystals with periodic boundary conditions were performed within the framework of the PAW method, as implemented in the VASP package [25,26,27,28] using the PBE functional and the Gamma-centered Brillouin zone. The plane-wave energy cut-off was set to 400 eV throughout the geometry optimization. Grimme D3 dispersion correction was applied [29]. The initial atomic coordinates for optimization and the unit cell parameters were obtained from experimental X-ray data. The optimized geometries were used for the accurate calculation of the electronic density (the energy cut-off was set to 500 eV), and further analysis was carried out in the framework of QT-AIM theory [30] using the AIM-UC package [31] to find the critical points and bonding paths. The energies of the coordination bonds and H-bonds were estimated as ¹⁄₂V(r_CP_) by Espinosa-Lecomte correlation for the closed-shell interaction, where V(r_CP_) is a potential energy density in the bond critical point [32].

## 3. Discussion

### 3.1. Synthesis

In order to search for new lanthanide complexes to be employed as luminescent thermometers, we embarked on the investigation of a pivalate platform which can produce a great variety of the possible architectures due to the unique steric properties of the pivalate ligand. Lanthanide pivalates and their mixed-ligand complexes have been studied in great detail in the literature [33,34,35], as well as in our group [21,36,37] for many years. The mixed-ligand complexes Ln(piv)_3_(en) were synthesized by the direct reaction of Ln(piv)_3_ with ethylenediamine in acetonitrile-benzene medium. One of the features of lanthanide tris-pivalates reported earlier is their crystallographic instability for the middle of the lanthanide series (Pr–Gd), as the bulky tert-butyl groups of pivalate ligands obstruct the step-wise change of the coordination mode along the series, in contrast to the lanthanide acetates [38,39,40,41,42]. This leads to the low crystallinity of the corresponding tris-pivalates due to the dynamic nature of pivalate ligand coordination modes at room temperature.

**Figure 1:**
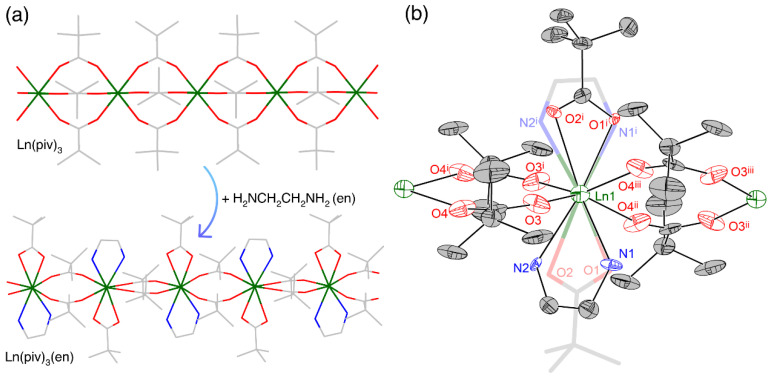
(**a**) Transformation of the Ln(piv)_3_ coordination polymer into the Ln(piv)_3_(en) coordination polymer. Note the alternating motif of the disordered chelating ligands in Ln(piv)_3_(en) with the alternative ligand arrangement depicted as a stick model with pale lines. (**b**) Crystal structure of Eu_0.53_Tb_0.47_(piv)_3_(en). Symmetry codes: (i) −x, −y, z; (ii) x, −y, 0.5 + z; (iii) −x, y, 0.5 + z.

**Figure 2:**
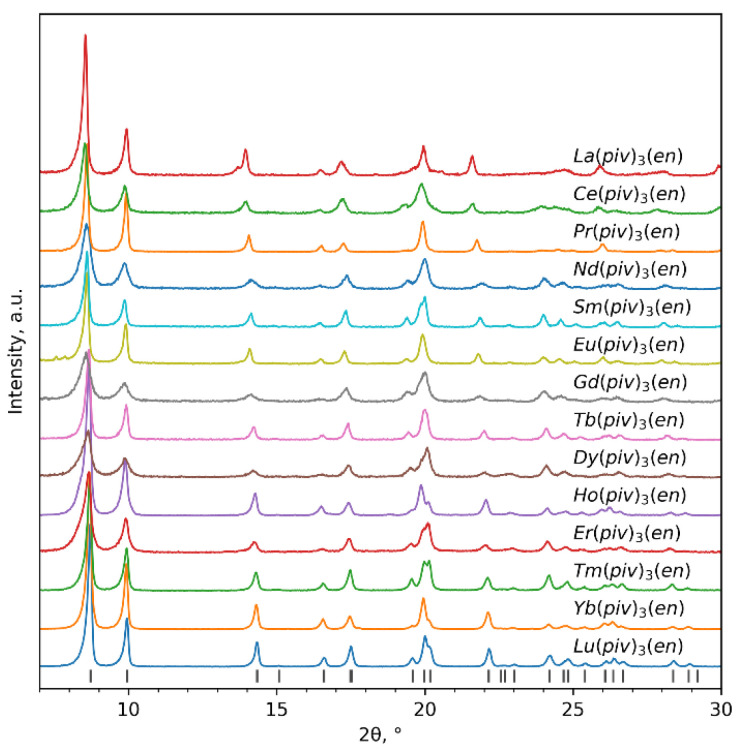
PXRD patterns of Ln(piv)_3_(en). The bars at the bottom represent calculated peak positions from single-crystal XRD data of the Eu_0.53_Tb_0.47_(piv)_3_(en) complex. λ = 1.5406 Å.

The direct reaction of Ln(piv)_3_ with ethylenediamine leads to its transformation into the mixed-ligand complex (Figure 1a and Figure S2) and the appearance of additional chelating ligands in the coordination sphere. The presence of two chelating ligands and two bridging ligands instead of three bridging ligands in Ln(piv)_3_ allows the crystal structure to adapt and better accommodate the changing ionic radii along the lanthanide series. This together with the steric properties of bulky pivalate ions helps to maintain the similar crystal structure and coordination environment for a full range of lanthanides (Figure 2). The unit cell parameters *a* and *c* change gradually (Figure S3a,c) according to the adjustment of the coordination sphere: parameter *a* decreases due to the shortening of the Ln–chelating ligand distance, and parameter *c* decreases due to shrinkage of the chain. Parameter *b* is not subject to any significant change (Figure S3b), as it corresponds to the van der Waals contacts of bridging pivalate ligands.

The predictability of the coordination environment of metal atoms is essential for the applications of lanthanide compounds, as the coordination environment significantly affects the pathways of energy dissipation and thus the luminescent properties of the complexes. Furthermore, the fact that the isostructural complexes exist for the whole lanthanide series offers the prospect of creating a wider range of heterometallic complexes based on the Ln(piv)_3_(en) structure type. The importance of such complexes lies in the possible fine-tuning of the energy transferred between different lanthanide atoms within a complex, as required for a wide range of luminescent materials with conventional [17,20,43,44] and up-conversion [45,46,47,48] fluorescence. 

### 3.2. Thermal Behavior

**Figure 3:**
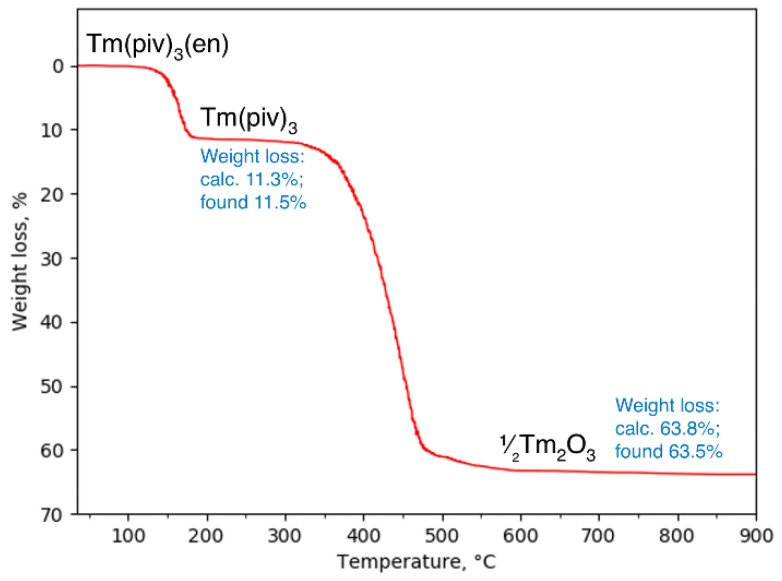
TG curve of Tm(piv)_3_(en).

Ln(piv)_3_(en) complexes are stable in air up to ca. 150 °C (Figure 3 and Figure S1). At higher temperatures, ethylenediamine is eliminated and Ln(piv)_3_ is formed, which is stable up to 300–400 °C, depending on the lanthanide. The temperature of ethylenediamine departure correlates with a mixed-ligand complex stability toward Ln(piv)_3_ formation. Previously, we reported on the stability of Ln(piv)_3_ along the series, and elucidated that it gradually increases along the lanthanide series [21]. This is in line with the current data on Ln(piv)_3_(en) thermal behavior, which reveal that the temperature of ethylenediamine departure decreases along the lanthanide series (Table S1), indicating the higher stability of the formed Ln(piv)_3_ for the heavier lanthanides.

### 3.3. X-ray Crystallography

The crystal structures of Eu_0.53_Tb_0.47_(piv)_3_(en), Tm(piv)_3_(en), and Lu(piv)_3_(en) were determined by means of X-ray diffraction. The orthorhombic unit cell (*Iba*2 space group) of Ln(piv)_3_(en) contains one crystallographically unique Ln atom (Figure 1b) coordinated by four symmetry-related pivalate anions (O3, O4 and their equivalents) bridging the Ln atoms and producing infinite 1D chains propagating along crystallographic axis *c*. The Ln atoms are also coordinated by a chelating pivalate (O1, O2) and a chelating ethylenediamine (N1, N2), with both being statically disordered over two symmetry-related positions with and equal occupancy factor of 0.5. One could expect the alternating ordering of the chelating ligands along the 1D polymeric chain (Figure 1a) due to sterical reasons. 

The coordination number of Ln is 8, and its coordination polyhedron is best described as a distorted biaugmented trigonal prism, according to the continuous shape measures (CShM) approach (Full list of CShM values is given in Table S2) [49]. Selected interatomic distances in the complexes are listed in Table 2.

Disordered {Ln(piv)(en)} fragments are connected by four bridging pivalate ligands into a 1D polymeric chain along crystallographic direction *a* (Figure S7). The uncertainty of the ligand arrangement in this partially ordered crystal structure stems from the similar size and bite angle of ethylenediamine and pivalate, and from the absence of any specific intermolecular interactions between ribbons, which is further confirmed by periodic DFT calculations. 

The systematic disordering of chelating ligands leads to the appearance of pseudo-symmetry, and makes it impossible to reveal more structural details from only Bragg scattering data. In principle, additional details could be manifested in the total (Bragg + diffuse) X-ray scattering pattern and pair distribution function (PDF). This method proved to be useful for the study of the local structure of poorly crystalline materials in solid form and in solution [50,51].

**Figure 4:**
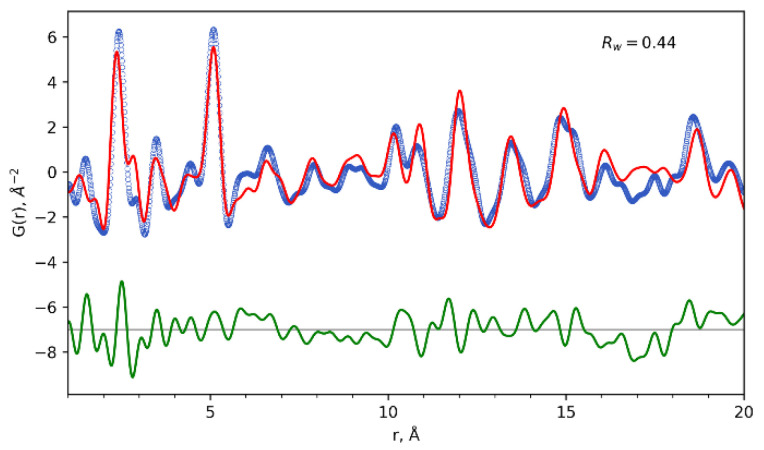
PDF fits (red solid line) of the experimental data (blue circles) of Eu_0.53_Tb_0.47_(piv)_3_(en) for the distance range 1–20 Å. The difference curve (green) is offset for clarity.

Therefore, we performed total scattering experiments with PDF analysis for Eu_0.53_Tb_0.47_(piv)_3_(en) powder (Figure 4). The data were collected on a STOE STADI P diffractometer equipped with Ag anode [52]; PDF calculations were done with PDFgetX3 [53] and refinements were carried out with DiffPy-CMI [54] (See Supplementary Materials for details). For the PDF refinements, the model was set up as a fragment of the Eu_0.53_Tb_0.47_(piv)_3_(en) crystal structure containing the seven closest polymeric chains, with nine metal atoms in each one. The experimental data are well described by this model, without further refinement of its structural parameters (atomic coordinates and atomic displacement parameters).

Several considered structural models (Figures S8 and S9) with different chelating ligand ordering (ethylenediamine and pivalate) or chain orientation give almost identical fits to the experimental PDF data (Figures S10 and S11). Therefore, the observed disorder does not stem from the limitations of single-crystal X-ray diffraction but rather is a specific feature of the studied coordination polymers.

### 3.4. Periodic DFT Calculations

**Figure 5:**
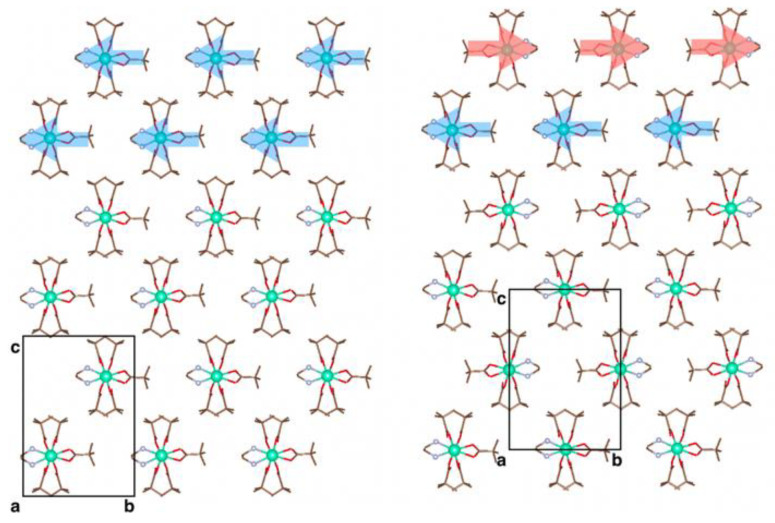
Two packing models of Lu(piv)_3_(en) considered in periodic DFT calculation: with *Pca*2_1_ (**left**) and *Ia* (**right**) symmetry. View along the polymeric chain axis. The black boxes are the unit cell projection. Colored arrows highlight different motifs of the chain packing.

We performed periodic DFT calculations to support the findings of the X-ray diffraction and to gain a deeper insight into whether there is a preferential way of packing of the 1D polymeric chains. According to the X-ray diffraction data, there are two distinct inter-chain contact directions within a plane orthogonal to the chain axis: one corresponding to contacts of pivalate anions only (crystallographic direction *c*), and another corresponding to contacts of pivalates and ethylenediamine (crystallographic direction *b*) (Figure 5). We considered an alternating motif of ligand arrangement within each chain and built two packing models with different relative orientations of chains, resulting in space groups *Pca*2_1_ and *Ia*, both being in line with the X-ray diffraction data. Geometry optimization with fixed unit cell parameters was performed for the La, Gd, and Lu complexes as representatives of the beginning, the middle, and the end of the lanthanide series, respectively.

The calculated Ln–O and Ln–N interatomic distances in the optimized models are similar to the experimental ones obtained from the XRD data (Table S3).

The resulting energy difference (ΔE) between two packing models is infinitesimally small (Table 3). This is in line with the findings of the X-ray diffraction indicating no preferential packing motif among the two considered options. The absence of a long-range order along a particular direction in the crystal structures containing bulky pivalate anions is a common feature of such systems, and the examples were reported earlier by our group [21,55].

### 3.5. Bonding Analysis

The analysis of Ln–O and Ln–N, and the H-bond bonding energies in the Ln(piv)_3_(en) compounds was performed within the framework of the Atoms-in-Molecules (AIM) approach by the evaluation of the Laplacian ∇^2^ρ of the calculated electron density distribution ρ, and by finding the (3, −1) critical points (Bond Critical Points, BCP) (Table 4, Figure S12). Four entities were considered for the calculation in accordance with the obtained DFT lattice energy values (Table 3): La(piv)_3_(en) in the *Ia* space group, Gd(piv)_3_(en) in the *Pca*2_1_ space group, and Lu(piv)_3_(en) in the *Pca*2_1_ space group.

The obtained total bonding energy values are smaller than those of the corresponding Ln(piv)_3_ previously reported by our group (531.2, 649.9, 719.2 kJ/mol for La(piv)_3_, Gd(piv)_3_, and Lu(piv)_3_, respectively) [21]. At the same time, the bonding energies of the H-bonds clearly indicate their tangible contribution to the total bonding energy. This gives more insight into the possible ligand arrangement within the 1D chain, as only two of the three possible configurations allow the formation of the H-bonds. 

### 3.6. Luminescent Thermometry

The existence of isostructural Ln(piv)_3_(en) complexes for the whole lanthanide series is an advantage for the design of new luminescent materials, as it provides the possibility to include different lanthanides in the same structure with a regular coordination environment. The 1D architecture of the complexes reduces the number of neighbors of each lanthanide, thus making the pathways of energy transfer between the lanthanides more predictable.

The heterometallic mixed-ligand complex Eu_0.05_Tb_0.95_(piv)_3_(en) was prepared by the reaction between Eu_0.05_Tb_0.95_(piv)_3_ and en. The unit cell parameters of Eu(piv)_3_(en), Tb(piv)_3_(en) and Eu_0.05_Tb_0.95_(piv)_3_(en) change accordingly to Vegard’s law (Figure S4).

The excitation spectra of Ln(piv)_3_(en) and Ln(piv)_3_ for Ln = Eu, Tb were recorded at room temperature by monitoring the emission wavelength of 615 nm (^5^D_0_→^7^F_2_ of Eu^3+^) and 545 nm (^5^D_4_→^7^F_5_ of Tb^3+^) (Figure S13). The effective excitation of the corresponding emission bands for homometallic Ln(piv)_3_(en) occurs at 383 and 395 nm (^7^F_0_→^5^G_2,4,6_ and ^7^F_0_→^5^L_6_ of Eu^3+^) for the Eu compound, and at 359, 378 and 493 nm (^7^F_6_→^5^D_2_; ^7^F_6_→^5^D_3_,^5^G_6_ and ^7^F_6_→^5^D_4_ of Tb^3+^) for the Tb compound. For the heterometallic Eu_0.05_Tb_0.95_(piv)_3_(en), the excitation of the Tb^3+^ transitions at 359, 378 and 493 nm results in both Eu^3+^ (615 nm) and Tb^3+^ (545 nm) emission. This indicates the active Tb^3+^→Eu^3+^ energy transfer within the coordination polymer (Figure 4). Importantly, the mechanical mixture of Eu(piv)_3_(en) and Tb(piv)_3_(en) with the same metal ratio exhibits only pure excitation spectra of the corresponding Eu and Tb emission without Tb^3+^→Eu^3+^ energy transfer. On the other hand, Eu_0.05_Tb_0.95_(piv)_3_ also shows the activation of Eu^3+^ emission through Tb^3+^ excitation bands. However, in the absence of the quenching en ligand, Tb^3+^→Eu^3+^ energy transfer seems to be too active, and the Tb^3+^ emission is less intensive than that of Eu^3+^.

**Figure 6:**
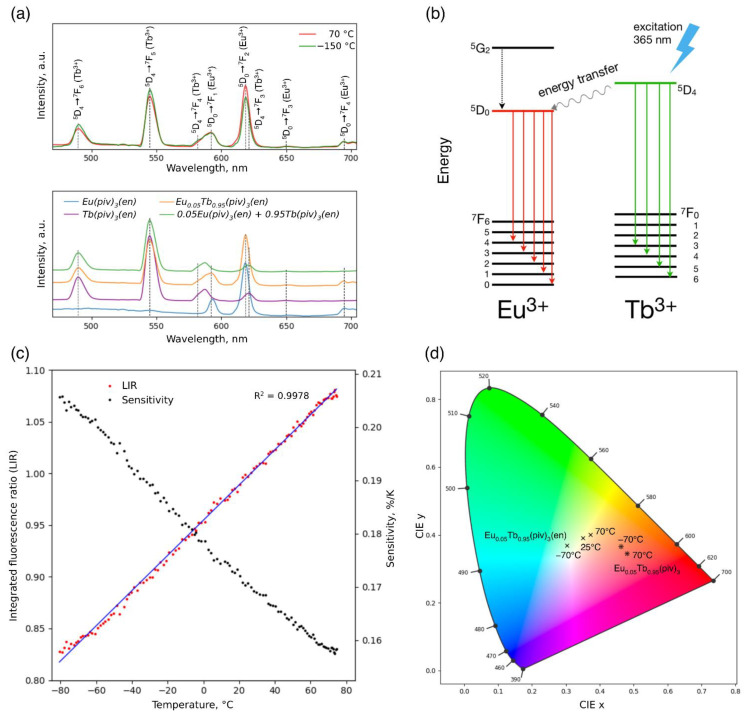
(**a**) Photoluminescence emission spectra of Eu_0.05_Tb_0.95_(piv)_3_(en) at high and low temperatures (**upper plot**); emission spectra of Ln(piv)_3_(en) (Ln = Eu, Tb, Eu_0.05_Tb_0.95_) at room temperature (**lower plot**). The vertical lines refer to the positions of emission peaks corresponding to the respective electron transitions. The excitation wavelength was 365 nm. See SI for the photoluminescence excitation spectra. (**b**) Schematic representation of the energy levels of Eu and Tb ions. (**c**) Temperature dependence of the integrated luminescent intensity ratio of the ^5^D_0_→^7^F_2_ transition of Eu^3+^ (615 nm) and the ^5^D_4_→^7^F_5_ transition of Tb^3+^ (545 nm) in Eu_0.05_Tb_0.95_(piv)_3_(en) (red markers), its linear fit (blue line), and the temperature dependence of the corresponding luminescent thermometer sensitivity (black markers). The excitation wavelength was 365 nm. (**d**) CIE 1931 chromaticity diagram with the coordinates of Eu_0.05_Tb_0.95_(piv)_3_(en) (×) and Eu_0.05_Tb_0.95_(piv)_3_ (❊) under 365 nm LED illumination at high and low temperatures. See Table S5 for the CIE x and y values.

The emission spectra of the compounds recorded at room temperature under an excitation of 365 nm LED demonstrate characteristic Tb and Eu luminescence due to ^5^D_0_→^7^F_J_ (Eu^3+^, J = 0–4) and ^5^D_4_→^7^F_J_ (Tb^3+^, J = 3–6) transitions (Figure 6a and Figure S14).

The temperature dependence of the Eu_0.05_Tb_0.95_(piv)_3_(en) emission spectra was recorded in the range of −150 °C to 80 °C (Figures S15 and S16). The compound demonstrates the change of the emission spectrum with temperature, and a color variation can be clearly observed (Figure 4 and Figure S17), which allows its application as a luminescent thermometer. 

The ratio of integrated intensities of the ^5^D_0_→^7^F_2_ (Eu^3+^) and ^5^D_4_→^7^F_5_ (Tb^3+^) transitions was used as a thermometric parameter (LIR, luminescence intensity ratio) which demonstrates linear dependence within the temperature range from −80 °C to 80 °C. For the precursor Eu_0.05_Tb_0.95_(piv)_3_ tris-pivalate, the luminescence measurements demonstrated the strong predominance of Eu luminescence (Figure S13). The introduction of ethylenediamine lead to the partial quenching of Eu luminescence, with more remarkable color variation (Figure 6d). The figure of merit used for the evaluation of a luminescent thermometer is its relative sensitivity, S_r_, expressed as S_r_ = 1/LIR(dLIR/dT). Eu_0.05_Tb_0.95_(piv)_3_(en) demonstrates a maximum sensitivity of 0.2%/K at the lower temperature of −80 °C (Figure 6c). A number of lanthanide-based luminescent thermometers with sensitivities up to 31%/K have been reported [18,19,56]. The aromatic organic ligands often employed to create the thermometers increase the sensitivity, as the energy transfer from ligand to metal is temperature sensitive. Consequently, the sensitivity values of the thermometers based on inorganic or non-aromatic matrices tend to be much lower [57,58]. Among these, the Eu_0.05_Tb_0.95_(piv)_3_(en) thermometer reported here demonstrates a comparable sensitivity value. At the same time, the clear benefits of the Ln(piv)_3_(en) platform are the wide range of LIR linearity for the reported Eu_0.05_Tb_0.95_(piv)_3_(en) and the existence of the same structure type for the whole lanthanide series, making it possible to create other heterometallic lanthanide-based materials for optical applications.

## 4. Conclusions

In summary, we have synthesized a new family of isostructural 1D polymeric mixed-ligand complexes Ln(piv)_3_(en) based on lanthanide pivalates for the whole lanthanide series. The introduction of ethylenediamine to Ln(piv)_3_ transforms the coordination environment but retains the 1D polymeric motif due to the presence of chelating ligands simplifying the adjustment of the ligand environment to the ionic radius of a particular lanthanide. 

Periodic DFT calculations revealed chain stabilization by intrachain H-bonds and weak interchain interactions. Noticeably, the energy difference was infinitesimally small (less than 7 kJ/mol) between the two considered packing polymorphs, leading to the partially ordered crystal structure. Pair distribution function analysis demonstrated that the observed disorder does not stem from the limitations of single-crystal X-ray diffraction but rather is a specific feature of the studied coordination polymers.

The luminescent properties of corresponding Eu and Tb complexes were investigated in a wide temperature range, and the possibility of energy transfer between lanthanides was confirmed. The prospect of creating the heterometallic complexes with inter-metal energy transfer for optical applications was demonstrated by the synthesis of Eu_0.05_Tb_0.95_(piv)_3_(en) and its investigation as a compound for luminescence thermometry. This demonstrated the linear temperature dependence of the thermometric parameter within the temperature range from −80 °C to +80 °C, and a maximum relative sensitivity value of 0.2%/K.

## Figures and Tables

**Table 1:** Crystallographic and refinement data for Ln(piv)_3_(en).

	Eu_0.53_Tb_0.47_(piv)_3_(en)	Tm(piv)_3_(en)	Lu(piv)_3_(en)
Formula	Eu_0.53_Tb_0.47_C_17_H_35_N_2_O_6_	TmC_17_H_35_N_2_O_6_	LuC_17_H_35_N_2_O_6_
Formula weight (g·mol^−1^)	518.72	532.4	538.4
Diffractometer	Bruker SMART APEX II	Rigaku SmartLab	Rigaku SmartLab
Wavelength (Å)	0.71073 (Mo Kα)	1.54187 (Cu Kα)	1.54187 (Cu Kα)
Data collection method	ω-scans	θ-θ scan	θ-θ scan
Temperature (K)	120	293	293
Crystal system	Orthorhombic	Orthorhombic	Orthorhombic
Space group	*Iba*2	*Iba*2	*Iba*2
*a* (Å)	12.329(10)	12.3746(3)	12.3443(2)
*b* (Å)	17.539(14)	17.7891(4)	17.7621(4)
*c* (Å)	10.134(8)	10.1367(3)	10.1222(5)
α (°)	90	90	90
β (°)	90	90	90
γ (°)	90	90	90
V (Å^3^)	2191(3)	2231.42(10)	2219.40(12)
Z	4	4	4
Color, habit	Colorless, needle	White, powder flat sheet	White, powder flat sheet
Sample dimensions (mm)	0.25 × 0.06 × 0.06	25 × 25 × 0.5	25 × 25 × 0.5
D_calc_ (g·cm^−^^3^)	1.572	1.5848	1.6114
μ (mm^−^^1^)	3.066	7.712	8.781
Unique reflections (R_int_)	3077(0.1249)	499	496
Observed reflections [*I* > 2σ(*I*)]	1667	499	496
Parameters, restrains	169, 65	83, 38	83, 38
*R*_1_[*I* > 2*σ*(*I*)], ωR_2_	0.0583, 0.1553	–	–
R_Bragg_, R_p_, ωR_p_	–	0.0152, 0.0350, 0.0526	0.0189, 0.0510, 0.0757
Goodness-of-fit ^1^	1.002	4.35	5.23
Absorption correction	SADABS	not required	not required
T_min_, T_max_	0.6202, 0.8039	–	–
ρ_min_, ρ_max_ (eÅ^−^^3^)	−2.609, 2.213	−1.43, 2.72	−1.43, 2.72

^1^ Goodness-of-fit is calculated on F^2^ for single-crystal diffraction data for [Eu_0.53_Tb_0.47_(piv)_3_(en)], and on the overall pattern intensities for powder diffraction data for [Tm(piv)_3_(en)] and [Lu(piv)_3_(en)].

**Table 2:** Selected interatomic distances (Å) and angles (°) in Ln(piv)_3_(en) crystal structures. Symmetry codes: (i) −x, −y, z; (ii) x, −y, 0.5 + z; (iii) −x, y, 0.5 + z; (iv) −x, y, −0.5 + z.

Parameter	Eu_0.53_Tb_0.47_(piv)_3_(en)	Tm(piv)_3_(en)	Lu(piv)_3_(en)
Ln1–O1	2.47(4)	2.41(3)	2.415(11)
Ln1–O2	2.58(2)	2.41(2)	2.415(11)
Ln1–N1	2.51(6)	2.45(3)	2.541(8)
Ln1–N2	2.46(5)	2.45(3)	2.541(8)
Ln1–O3	2.33(3)	2.208(13)	2.237(13)
Ln1–O3 ^i^	2.33(3)	2.208(13)	2.237(13)
Ln1-O4 ^ii^	2.32(3)	2.352(14)	2.199(12)
Ln1–O4 ^iii^	2.32(3)	2.352(14)	2.199(12)
Ln1⋯Ln1 ^iv^	5.067(8)	5.0684(3)	5.0611(5)
∠Ln1-O3-C6	141(2)	138.4(9)	158.7(7)
∠Ln1 ^iv^-O4-C6	167(2)	167.6(9)	153.2(7)

**Table 3:** DFT calculated lattice energies of two packing polymorphs of Ln(piv)_3_(en) in different space groups—*Pca*2_1_ and *Ia*—and their difference, ΔE = E(*Pca*2_1_) − E(*Ia*), per mole of unit cells. Negative values of ΔE correspond to a higher stability of the *Pca*2_1_ polymorph.

Compound	E(*Pca*2_1_), eV	E(*Ia*), eV	ΔE, kJ/mol
La(piv)_3_(en)	−1433.1727	−1433.1828	0.97
Gd(piv)_3_(en)	−1432.2695	−1432.2314	−3.68
Lu(piv)_3_(en)	−1431.7396	−1431.6679	−6.92

**Table 4:** Calculated bonding energies in DFT optimized Ln(piv)_3_(en) structures. The energies for the most stable polymorph (Table 3) are presented. The values presented in the columns correspond to sums of bonding energies with four atoms of the bridging ligands, four atoms of the chelating ligands, two H-bonds, and the total of the three, respectively. For the calculated electron densities and the bonding energies of the individual bonds, see Table S4.

	La(piv)_3_(en)	Gd(piv)_3_(en)	Lu(piv)_3_(en)
ΣE_brid_, kJ/mol	268.97	363.95	411.89
ΣE_chel_, kJ/mol	190.17	234.01	253.69
ΣE_H_, kJ/mol	34.53	34.53	34.59
ΣE_tot_, kJ/mol	493.67	632.49	700.17

## Data Availability

The primary data presented in this study are available on request from the corresponding author.

## Supplementary Materials

The following supporting information can be downloaded at: https://www.mdpi.com/article/10.3390/polym14163328/s1. Figure S1: Typical TG curves of Ln(piv)_3_(en) for Ln from different parts of the lanthanide series (Ln = Ce, Eu, Tm). Table S1: Temperatures (°C) of ethylenediamine departure from Ln(piv)_3_(en) (Ln = La, Pr, Nd, Sm, Eu, Tm, Lu) estimated from the TG curves as a temperature coordinate of the point corresponding to half of ethylenediamine weight loss. Figure S2: IR spectra of Eu_0.05_Tb_0.95_(piv)_3_ and Eu_0.05_Tb_0.95_(piv)_3_(en). Figure S3: Unit cell parameters (a, b, c) and cell volume (V) of [Ln(piv)_3_(en)], Ln = La − Lu obtained from a Le Bail fit of PXRD data at 293 K in *Iba*2 space group (α = β = γ = 90°) vs. the Shannon ionic radius (CN = 8) of Ln^3+^. Red solid lines show the linear fit of the dependencies with the corresponding equations. Figure S4: Crystallographic parameter a obtained from a Le Bail fit of the PXRD data vs. Eu mole fraction in the total metal content in the corresponding Eu_x_Tb_1−x_(piv)_3_(en). The blue line corresponds to the linear dependence of the lattice parameter (Vegard’s law). Figure S5: Room temperature powder XRD pattern (black circles) of [Tm(piv)_3_(en)], Rietveld refinement fit (red solid line), difference profile (lower green solid line), and positions of Bragg peaks (vertical bars). Figure S6: Room temperature powder XRD pattern (black circles) of [Lu(piv)_3_(en)], Rietveld refinement fit (red solid line), difference profile (lower green solid line), and positions of Bragg peaks (vertical bars). Figure S7: Bond angles between bridging pivalate ligands and metal atoms in crystal structure of Ln(piv)_3_(en). Note the relative position of the bridging ligands resulting in absence of an inversion center within the fragment. This leads to non-centrosymmetric space group *Iba*2 in the crystal structure. Chelating ligands are omitted for clarity. Table S2: Continuous Shape Measures (CShM) analysis for Ln polyhedra in Ln(piv)_3_(en). Figure S8: Alternating motif of chelating ligand arrangement within the 1D chain of Ln(piv)_3_(en). Figure S9: (a) One-sided motif of the chelating ligand arrangement within the 1D chain of Ln(piv)_3_(en); (b) segregated motif of the chelating ligand arrangement within the 1D chain of Ln(piv)_3_(en). Figure S10: PDF fits (red solid line) of the measured data (circles) with different 1D chain models of Eu(piv)_3_(en) for the distance range 1–10 Å. The difference curves are offset for clarity. Figure S11: PDF fits (red solid line) of the measured data (circles) with different 17-chain models of Eu(piv)_3_(en) for the distance range 1–30 Å (upper and middle plots). Difference PDF (lower plot) obtained as a difference of two calculated PDFs. Table S3: Selected interatomic distances (Å) and angles (°) in the optimized geometry of Ln(piv)_3_(en) in *Pca*2_1_, and *Ia* space groups and in the Lu(piv)_3_(en) crystal structure. Symmetry codes (i) −x, −y, z; (ii) x, −y, 0.5 + z; (iii) −x, y, 0.5 + z; (iv) −x, y, −0.5 + z. Table S4: Calculated electron densities of the bond critical points of Ln–L and H-bonds in DFT optimized Ln(piv)_3_(en) structures, and the corresponding bonding energies. Figure S12: Fragment of a Lu(piv)_3_(en) polymeric chain with the alternating ligand arrangement. The small olive balls represent bond critical points. Symmetry code: (i) −x, −y, z. Figure S13: Photoluminescence excitation spectra of the Eu(piv)_3_, Tb(piv)_3_, Eu_0.05_Tb_0.95_(piv)_3_, Eu_0.05_Tb_0.95_(piv)_3_(en), and mechanical mixture Tb(piv)_3_ and Eu(piv)_3_, monitored at the emission wavelength of 545 nm (Tb^3+^: ^5^D_4_→^7^F_5_) and 615 nm (Eu^3+^: ^5^D_0_→^7^F_2_). Figure S14: Photoluminescence emission spectra of the Eu_0.05_Tb_0.95_(piv)_3_ at high and low temperatures. Excitation wavelength 365 nm. Figure S15: Temperature dependence of the integrated luminescent intensity ratio of ^5^D_0_→^7^F_2_ transition of Eu^3+^ (615 nm) and the ^5^D_4_→^7^F_5_ transition of Tb^3+^ (545 nm) in Eu_0.05_Tb_0.95_(piv)_3_(en). Excitation wavelength 365 nm. Figure S16: Scheme of the experimental setup for the temperature dependence of photoluminescence emission spectra measurements. The sample powder is fixed by carbon tape on copper-plated sample stage together with a thin-wire K-type thermocouple (Tactual). The heating and cooling of the sample stage occurs due to the nitrogen gas flow of a certain temperature (Tsetpoint). The temperature of the gas flow from the liquid N_2_ boiler is regulated by a Gas Flow Heater with a PID thermocontroller attached directly to the thermoisolation of the optical chamber. The actual sample temperature and emission spectra in quasi real-time mode were stored on the PC for further processing and analysis. Figure S17: Color of the compounds under 365 nm LED illumination at high and low temperatures. Table S5: CIE color coordinates of Eu_0.05_Tb_0.95_(piv)_3_ and Eu_0.05_Tb_0.95_(piv)_3_(en) under 365 nm LED illumination at high and low temperatures.
