# Supplementary material for: Partially Ordered Lanthanide Carboxylates with a Highly Adaptable 1D Polymeric Structure
Source: Polymers (Basel). 2022 Aug 16;14(16):3328. doi: 10.3390/polym14163328 (PMC9414554; doi:10.3390/polym14163328)
Supplement: Supplementary file 1 [file polymers-14-03328-s001.zip › polymers-1848043-supplementary.pdf]

Partially Ordered Lanthanide Carboxylates with a Highly Adaptable 1D Polymeric Structure

Dimitry Grebenyuk [1], Mirijam Zobel [2] and Dmitry Tsymbarenko [1,*]

[1] Department of Chemistry, Lomonosov Moscow State University, Leninskie gory 1, 119991 Moscow, Russia

[2] Institute of Crystallography, RWTH Aachen University, Jägerstr. 17-19, 52066 Aachen, Germany;

* Correspondence: tsymbarenko@gmail.com or tsymbarenko@inorg.chem.msu.ru

Abstract: A new family of 14 isostructural $[Ln(piv)_3(en)]_\infty$ lanthanide pivalate (piv^-, 2,2-dimethylpropanoate) complexes with ethylenediamine (en) were synthesized by a topology-preserving transformation from 1D coordination polymers $[Ln(piv)_3]_\infty$. Crystal structures of the compounds were determined by single crystal and powder X-ray diffraction which demonstrated that despite the regular ligand arrangement within the chains, the latter are intricately packed within the partially ordered crystal since only two of four ligands are strictly bound by the translational symmetry. The peculiarities of the lanthanide coordination environment were explored by total X-ray scattering with pair distribution function analysis. Periodic DFT calculations revealed the chain stabilization by intrachain H-bonds and weak interchain interactions. Noticeably, the energy difference is infinitesimally small even between the two considered extreme packing polymorphs which is in line with the disturbed packing order of the chains. The luminescent properties of Eu and Tb complexes have been investigated to prove the energy transfer between lanthanide ions within the heterometallic complex. This opens a prospect for creating new materials for optical applications. Heterometallic compound $Eu_{0.05}Tb_{0.95}(piv)_3(en)$ was synthesized and found to demonstrate temperature-dependent luminescence with a linear dependence of the thermometric parameter I(Eu)/I(Tb) within the temperature range from –80 °C to 80 °C and a maximum relative sensitivity value of 0.2 %/K.

Table of Contents

**Synthesis**	**S3**
**X-ray crystallography**	**S4**
**Pair distribution function analysis**	**S7**
**Periodic DFT calculations**	**S9**
**Bonding analysis**	**S9**
**Photoluminescence spectroscopy**	**S11**
**References**	**S14**

Synthesis

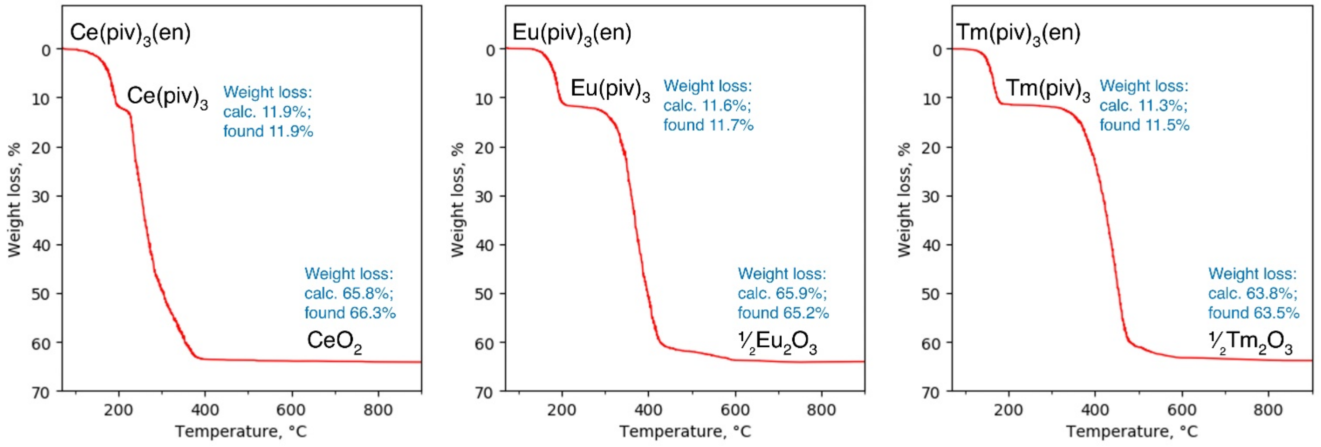

Figure S1. Typical TG curves of $Ln(piv)_3(en)$ for Ln from different part of the lanthanide series (Ln = Ce, Eu, Tm).

Table S1. Temperatures (°C) of ethylenediamine departure from $Ln(piv)_3(en)$ (Ln = La, Pr, Nd, Sm, Eu, Tm, Lu) estimated from the TG curves as a temperature coordinate of the point corresponding to half of ethylenediamine weight loss.

	La	Pr	Nd	Sm	Eu	Tm	Lu
$T_{departure}$, °C	188	185	195	193	185	162	151

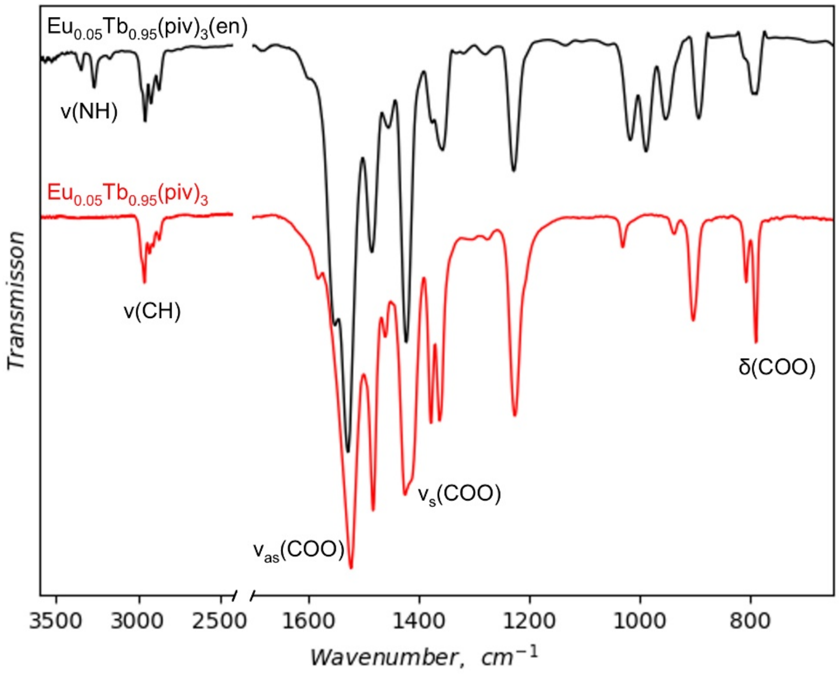

Figure S2. IR spectra of $Eu_{0.05}Tb_{0.95}(piv)_3$ and $Eu_{0.05}Tb_{0.95}(piv)_3(en)$.

X-ray crystallography

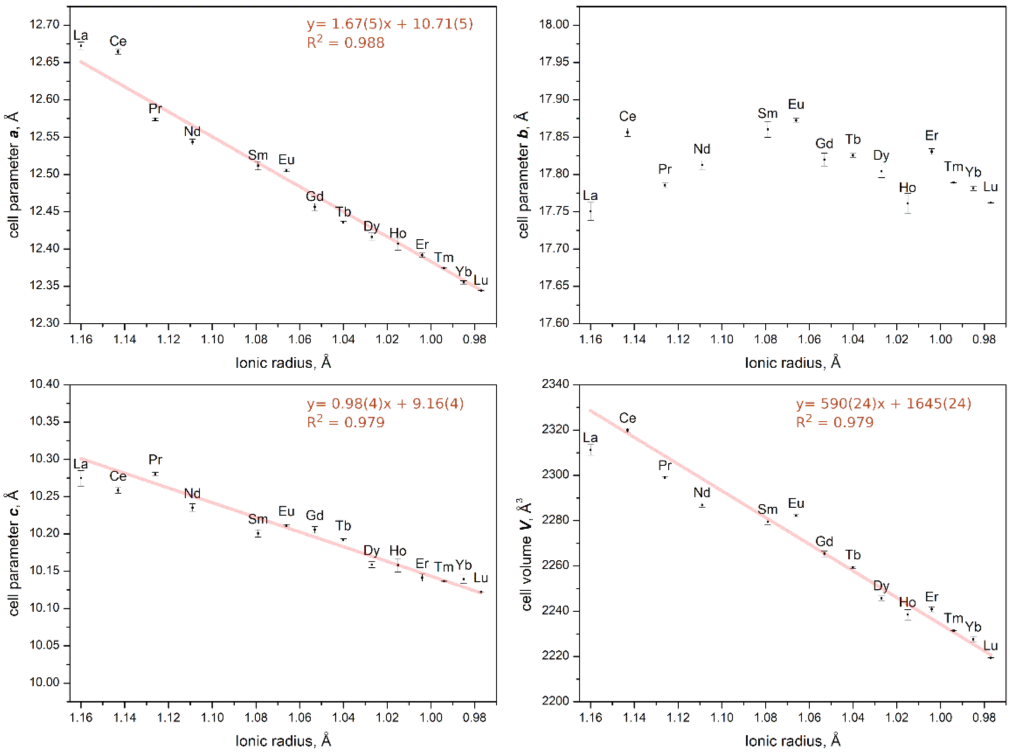

Figure S3. Unit cell parameters (*a, b, c*) and cell volume (V) of [Ln(piv)$_3$(en)], Ln = La–Lu obtained from Le Bail fit of PXRD data at 293 K in *Iba*2 space group ($\alpha = \beta = \gamma = 90^o$) vs Shannon ionic radius (CN = 8) of Ln^{3+}. Red solid lines show the linear fit of the dependencies with the corresponding equations.

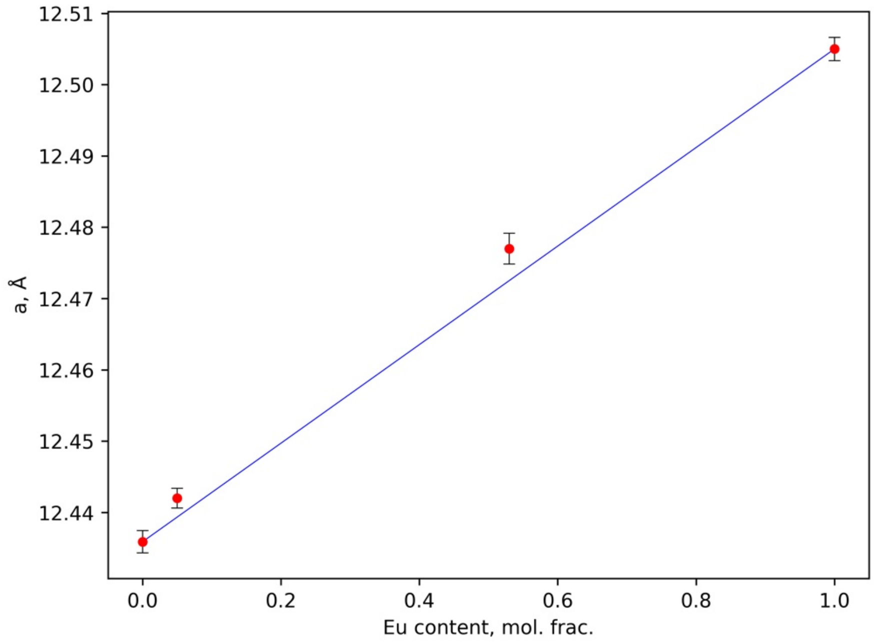

Figure S4. Crystallographic parameter *a* obtained from Le Bail fit of PXRD data vs Eu mole fraction in total metal content in the corresponding Eu_xTb_{1-x}(piv)$_3$(en). Blue line corresponds to the linear dependence of the lattice parameter (Vegard's law).

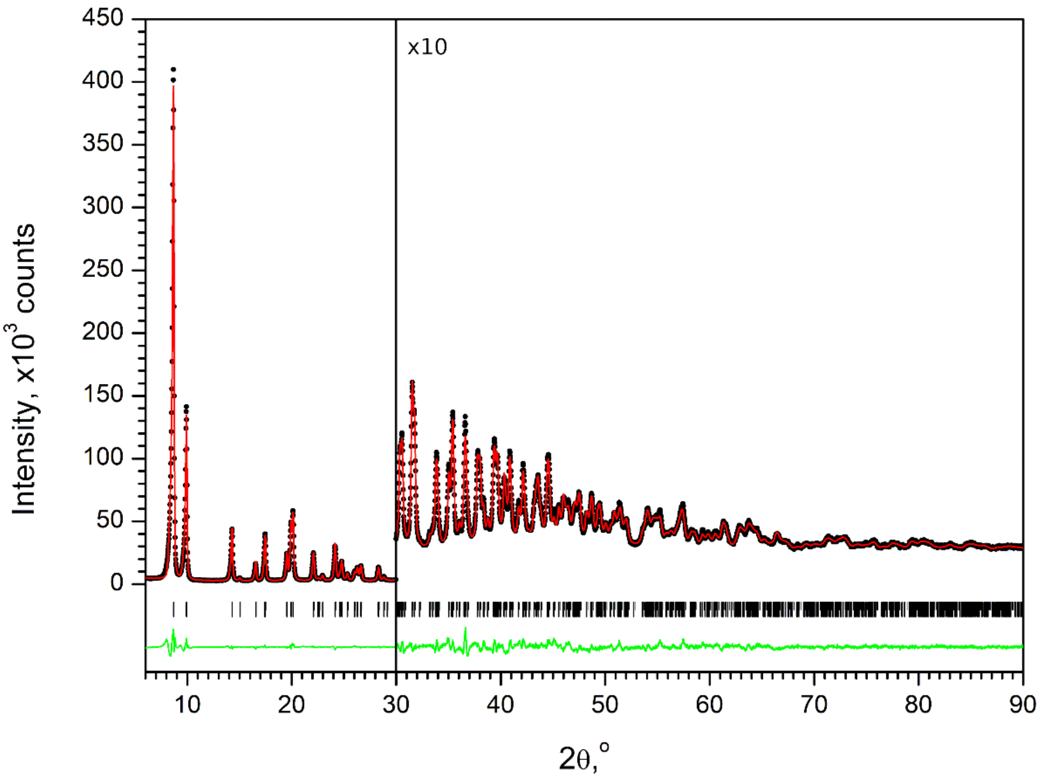

Figure S5. Room temperature powder XRD pattern (black circles) of [Tm(piv)$_3$(en)], Rietveld refinement fit (red solid line), difference profile (lower green solid line), and positions of Bragg peaks (vertical bars).

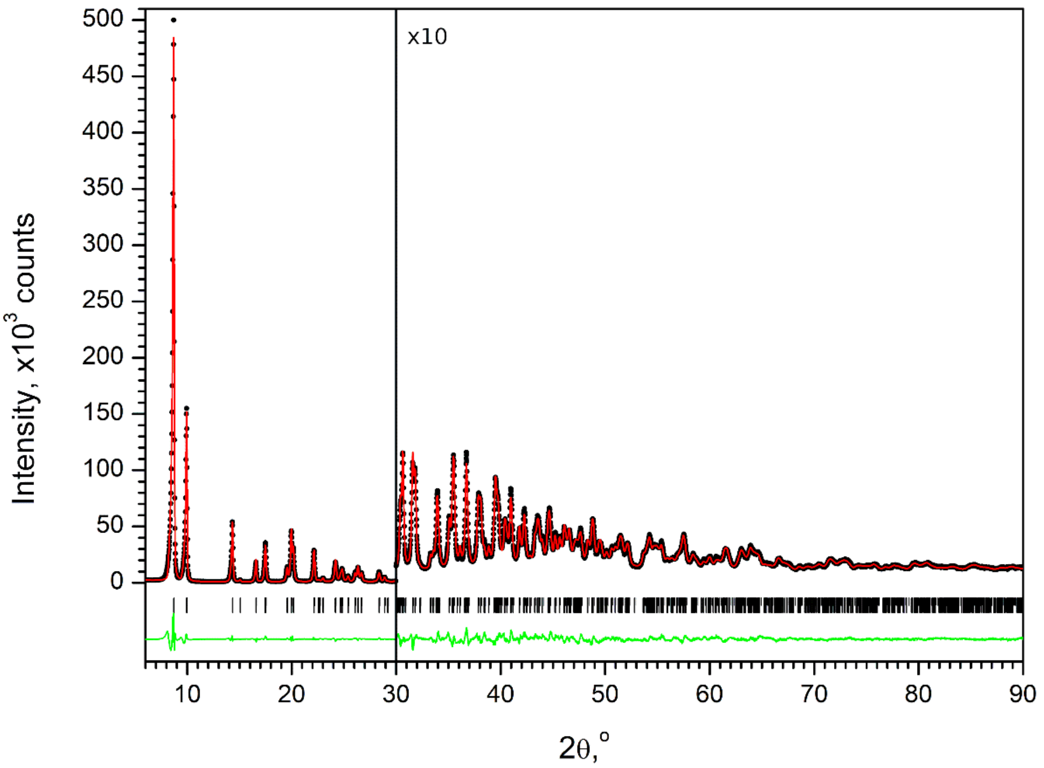

Figure S6. Room temperature powder XRD pattern (black circles) of [Lu(piv)$_3$(en)], Rietveld refinement fit (red solid line), difference profile (lower green solid line), and positions of Bragg peaks (vertical bars).

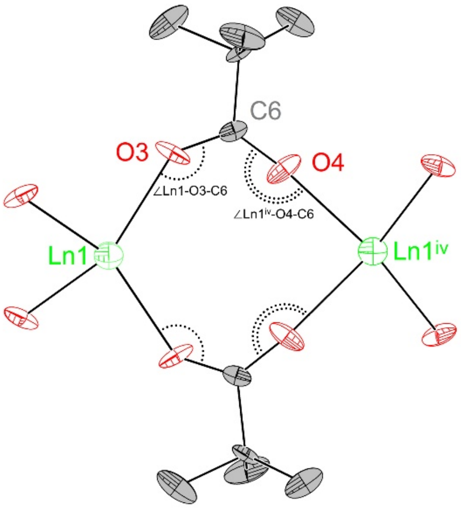

Figure S7. Bond angles between bridging pivalate ligands and metal atoms in crystal structure of $Ln(piv)_3(en)$. Note the relative position of the bridging ligands resulting in absence of an inversion center within the fragment. This leads to non-centrosymmetric space group Iba2 in the crystal structure. Chelating ligands are omitted for clarity.

Table S2. Continuous Shape Measures (CShM) analysis[1] for Ln polyhedra in $Ln(piv)_3(en)$.

OP-8	HPY-8	HBPY-8	CU-8	SAPR-8	TDD-8	JGBF-8	JETBPY-8	JBTPR-8	BTPR-8	JSD-8	TT-8	ETBPY-8
$Eu_{0.53}Tb_{0.47}(piv)_3(en)$												
29.116	22.929	13.242	11.203	3.413	2.855	11.658	26.277	2.191	1.535	3.033	11.886	23.857
$Tm(piv)_3(en)$												
28.661	21.039	14.342	12.393	4.241	3.414	11.454	25.589	2.761	1.981	3.571	13.087	23.038
$Lu(piv)_3(en)$												
28.665	21.269	13.625	13.173	3.753	2.671	9.794	25.639	2.966	2.999	2.648	13.800	22.717

Reference shapes: OP-8 — octagon; HPY-8 — heptagonal pyramid; HBPY-8 — hexagonal bipyraid; CU-8 — cube; SAPR-8 — square antiprism; TDD-8 — triangular dodecahedron (D_{2d}); JGBF-8 — Johnson gyrobifastigium J26 (D_{2d}); JETBPY-8 — Johnson elongated triangular bipyramid J14 (D_{3h}); JBTPR-8 — biaugmented trigonal prism J50 (C_{2v}); BTPR-8 — biaugmented trigonal prism (C_{2v}); JSD-8 — snub disphenoid J84 (D_{2d}); TT-8 — triakis tetrahedron; ETBPY-8 — elongated trigonal bipyramid (D_{3h}).

Pair distribution function analysis

Total X-ray scattering data for pair distribution function (PDF) analysis were collected in the moving mode on the STOE STADI P diffractometer in Debye-Scherrer geometry with a curved Ge(111) monochromator yielding AgKα1 radiation (λ=0.5594 Å) from a standard sealed tube (Malvern Panalytical C-Tech, Ag, 2.2 kW) in the 2θ range of 0.6-132° (Q range 0.1–20.5 Å-1) Dectris MYTHEN 4K detector was employed to collect the scattering data.[2] The sample was measured in 0.5 mm borosilicate glass capillaries. PDF calculations were done with PDFgetX3[3] using the Q-range of 0.8 to 13.8 Å^{-1} and refinements were carried out with DiffPy-CMI.[4] Atomic coordinates were taken from the crystal structure and were fixed during the fit. Fixed isotropic atomic displacement parameters were assigned as follows: Uiso(Ln) = 0.02, Uiso(O) = Uiso(N) = Uiso(C) = 0.03. The instrumental resolution parameters, peak broadening and damping were kept constant based on a calibration with a standard.[2] The scale factor and quadratic peak broadening parameter have been refined during the fit.

According to the single-crystal and powder X-ray diffraction data, the chelating ligands — pivalate anions and ethylenediamine molecules — are disordered over two symmetry-related positions with equal occupancy factor of 0.5. In principle, one could suppose the alternating motif of the ligand arrangement (Figure S8) due to the repulsion of bulky pivalates and possible intra-chain H-bonds between pivalate ions and amine molecules.

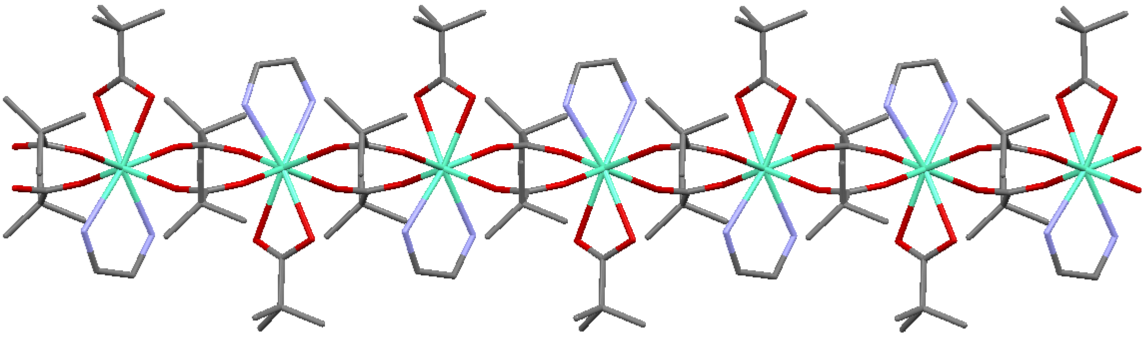

Figure S8. Alternating motif of chelating ligand arrangement within the 1D chain of Ln(piv)$_3$(en).

Trying to elucidate whether this is the case for Ln(piv)$_3$(en), and also how the make-up of the chain would affect the PDF, we performed the refinements of experimental PDF data with two different models: (1) one-sided with ligands of each type located on one side of the 1D chain (Figure S9a) and (2) segregated with two pivalate ligands coordinated to one metal atom alternating with two ethylenediamine ligands coordinated by the neighbour metal atom. It should be explicitly noted that all the models of 1D chains for the PDF refinements were constructed from the results of X-ray structural analysis and are consistent with the experimental diffraction data when the corresponding symmetry elements are applied.

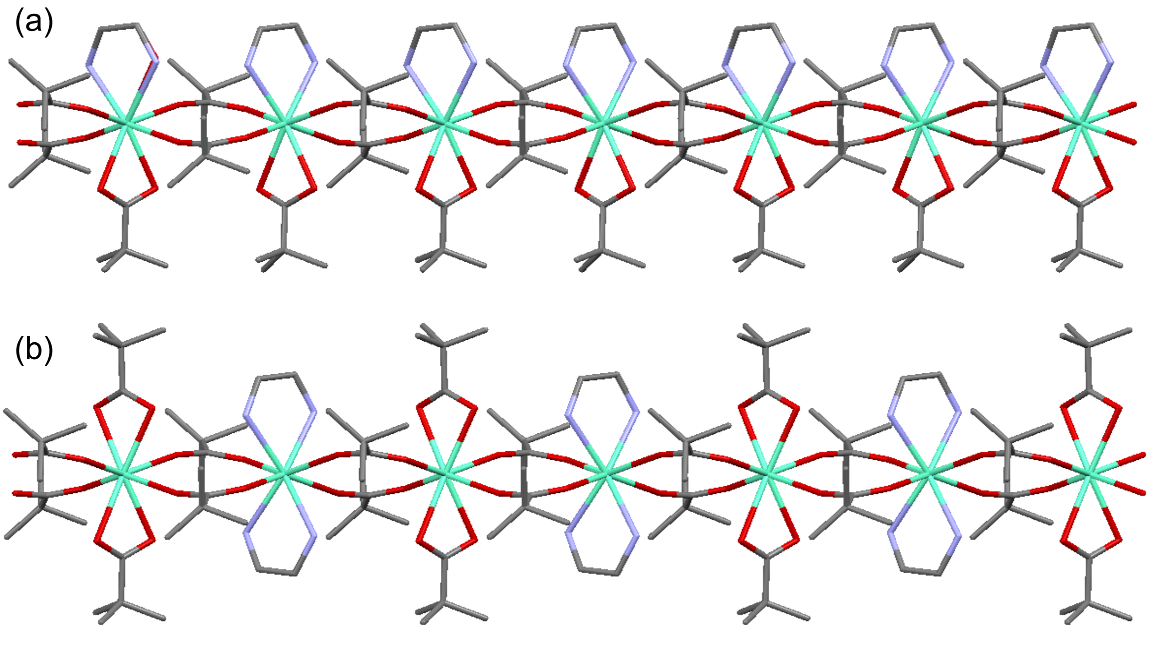

Figure S9. (a) One-sided motif of chelating ligand arrangement within the 1D chain of Ln(piv)$_3$(en); (b) segregated motif of chelating ligand arrangement within the 1D chain of Ln(piv)$_3$(en).

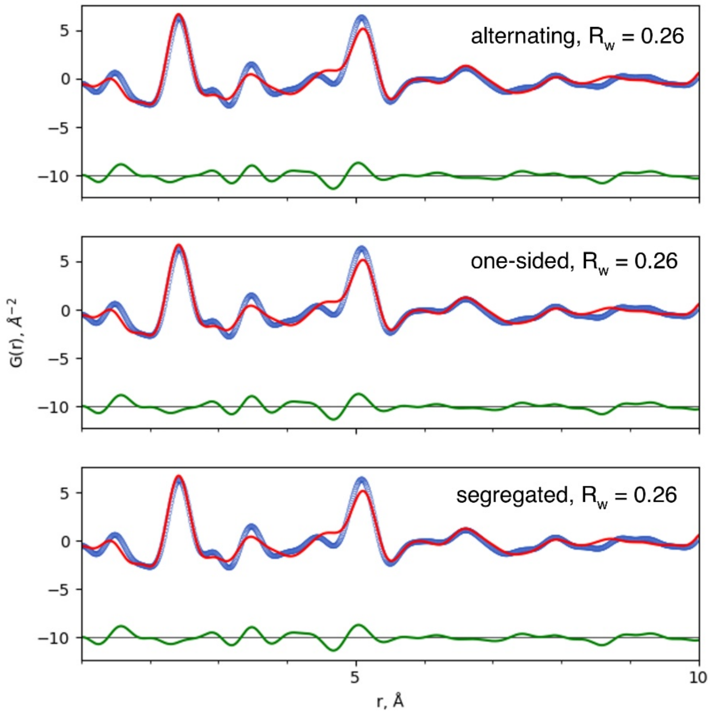

Figure S10. PDF fits (red solid line) of measured data (circles) with different 1D chain models of Eu(piv)3(en) for the distance range 1–10 Å. Difference curves are offset for clarity.

Results of the PDF refinements (Figure S10) demonstrate that the considered variants of the chain make-up do not impact the local structure enough for the difference to be manifested in the short-range of the calculated PDF. Therefore, we performed the refinements for the new structural models constructed from 17 identical chains (1 chain + group of 6 chains closest to it + group of 10 chains closest to the second group) with different relative arrangement, see Figure S11 top and middle panels. No difference in fit quality could be observed over this extended range of 1–30 Å either.

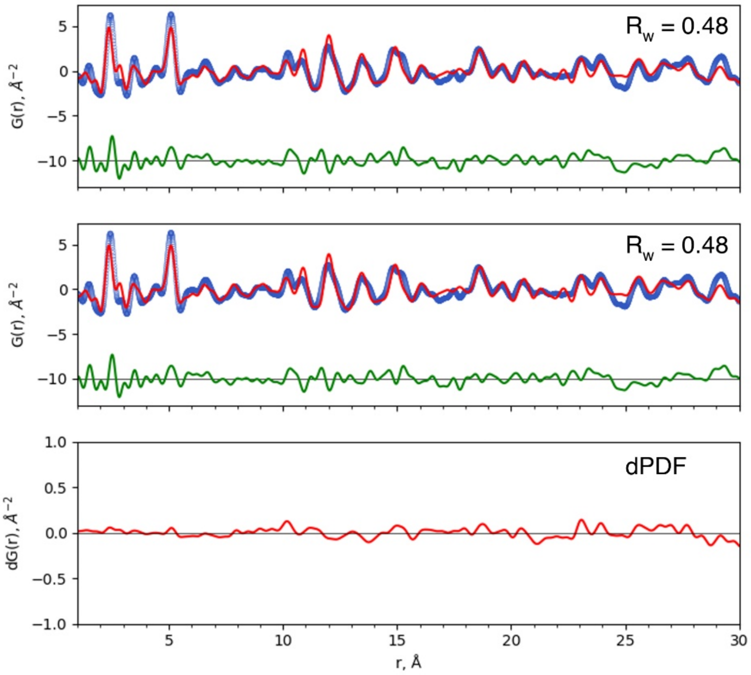

Figure S11. PDF fits (red solid line) of measured data (circles) with different 17-chain models of Eu(piv)$_3$(en) for the distance range 1–30 Å (upper and middle plots). Difference PDF (lower plot) obtained as a difference of two calculated PDFs.

Therefore, in order to highlight the structural differences of the two variants of 17-chain models, we calculated theoretical PDFs of both models over 30 Å and subtracted one from another. The resulting difference PDF (dPDF) is shown in Figure S11 bottom panel. Apparently, the scattering power of the light atoms (C, N, O, H) whose arrangement is different within the models is not high enough for this difference to be manifested in the PDFs. Therefore, total X-ray scattering technique does not shed the light on this kind of ligand disorder in Ln(piv)$_3$(en), though it independently confirms the findings of X-ray diffraction regarding the crystal structure of the complex.

Periodic DFT calculations

Table S3. Selected interatomic distances (Å) and angles (°) in optimized geometry of Ln(piv)$_3$(en) in *Pca*2$_1$, and *Ia* space groups and in Lu(piv)$_3$(en) crystal structure. Symmetry codes (i) -x, -y, z; (ii) x, -y, 0.5+z; (iii) -x, y, 0.5+z; (iv) -x, y, -0.5+z.

Parameter	Lu(piv)$_3$(en) in *Pca*2$_1$	Lu(piv)$_3$(en) in *Ia*	Lu(piv)$_3$(en) crystal structure
Ln1–O1	2.370	2.372	2.415(11)
Ln1–O2	2.412	2.415	2.415(11)
Ln1–N1	2.498	2.498	2.541(8)
Ln1–N2	2.513	2.519	2.541(8)
Ln1–O3	2.295	2.299	2.237(13)
Ln1–O3^{i}	2.276	2.285	2.237(13)
Ln1-O4^{ii}	2.217	2.195	2.199(12)
Ln1–O4^{iii}	2.200	2.222	2.199(12)
Ln1···Ln1^{iv}	5.061	5.061	5.0611(5)
∠Ln1-O3-C6	164.7	169.3	158.7(7)
∠Ln1^{iv}-O4-C6	145.5	141.6	153.2(7)

Bonding analysis

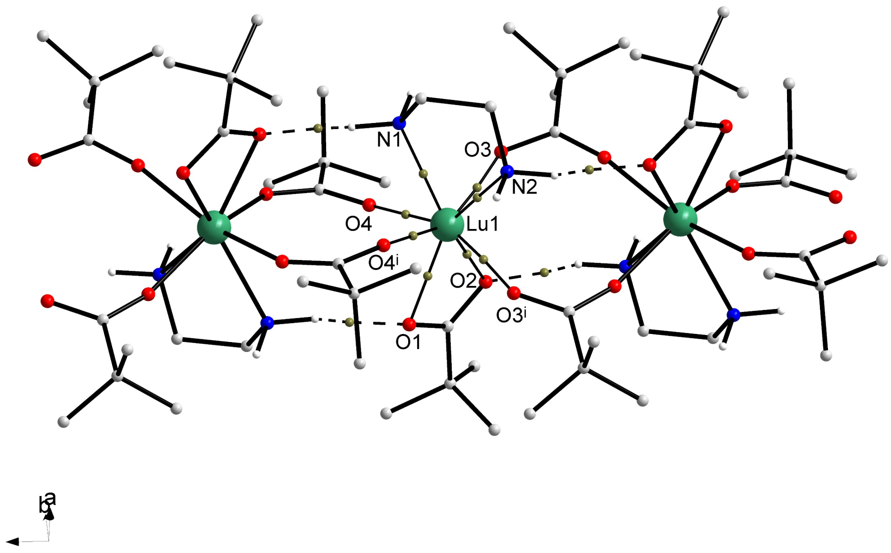

Figure S12. Fragment of a Lu(piv)$_3$(en) polymeric chain with the alternating ligand arrangement. Small olive balls represent bond critical points. Symmetry code: (i) -x, -y, z.

Table S4. Calculated electron densities of the bond critical points of Ln–L and H-bonds in DFT optimized $Ln(piv)_3(en)$ structures and the corresponding bonding energies.

	ρ, e · Å^{-3}	∇^2ρ, e · Å^{-5}	V, eV	E, kJ/mol	ΣE, kJ/mol*
			$Gd(piv)_3(en)$ in *Ia* space group		
Ln–O4	0.397493	6.05913	-0.072158207	94.79	
	0.4277504	6.374646	-0.079909092	104.97	
Ln–O3	0.3761109	4.930487	-0.063747385	83.74	
	0.3614479	4.907296	-0.060672539	79.70	363.20
Ln–N1	0.2827821	2.942005	-0.039204057	51.50	
Ln–N2	0.2893227	2.874966	-0.040099949	52.68	
Ln–O1	0.3095259	3.735817	-0.046667734	61.30	
Ln–O2	0.3330203	3.929846	-0.051715361	67.93	233.42
N1–H1a···O2	0.1309249	1.641573	-0.013720573	18.02	
N2–H2a···O1	0.1233508	1.532087	-0.012581437	16.53	34.55
			$Gd(piv)_3(en)$ in *Pca*2_1 space group		
Ln–O4	0.4189138	6.279542	-0.077601631	101.94	
	0.4055902	6.166443	-0.074279566	97.58	
Ln–O3	0.3623791	4.865311	-0.060715171	79.76	
	0.377885	5.030486	-0.064460877	84.68	363.95
Ln–N1	0.2850791	2.862439	-0.039323002	51.66	
Ln–N2	0.2891006	2.863338	-0.040021164	52.57	
Ln–O1	0.3329987	3.936693	-0.051734917	67.96	
Ln–O2	0.3114085	3.751155	-0.047063583	61.82	234.01
N1–H1a···O2	0.1259419	1.585271	-0.013022123	17.11	
N2–H2a···O1	0.1278759	1.599106	-0.013263935	17.42	34.53
			$Lu(piv)_3(en)$ in *Pca*2_1 space group		
Ln–O4	0.4327817	7.323182	-0.084327943	110.78	
	0.453054	7.644452	-0.090116912	118.38	
Ln–O3	0.3987349	5.992569	-0.072194957	94.84	
	0.378333	5.711706	-0.066909562	87.89	411.89
Ln–N1	0.2970456	2.936938	-0.041667846	54.74	
Ln–N2	0.3071613	3.159759	-0.044247118	58.12	
Ln–O1	0.318959	4.083926	-0.049603085	65.16	
Ln–O2	0.3522883	4.548897	-0.057603002	75.67	253.69
N1–H1a···O2	0.1273541	1.595451	-0.013198768	17.34	
N2–H2a···O1	0.1267157	1.595265	-0.013134054	17.25	34.59
			$La(piv)_3(en)$ in *Ia* space group		
Ln–O4	0.3833951	5.010651	-0.065541642	86.10	
	0.3495554	4.763611	-0.057805495	75.93	
Ln–O3	0.3267703	3.793262	-0.050057974	65.76	
	0.3398033	3.755128	-0.031348545	41.18	268.97
Ln–N1	0.2500744	2.112103	-0.030956585	40.67	
Ln–N2	0.2514788	2.16131	-0.031348545	41.18	
Ln–O1	0.2848546	2.969563	-0.039654826	52.09	
Ln–O2	0.3001379	3.107207	-0.042805272	56.23	190.17
N1–H1a···O2	0.1357007	1.700028	-0.014417674	18.94	
N2–H2a···O1	0.1306376	1.618086	-0.013609957	17.88	34.53

* Three values are presented in the column corresponding to sums of bonding energies with four atoms of the bridging ligands, four atoms of the chelating ligands and two H-bonds, respectively.

Photoluminescence spectroscopy

Photoluminescence excitation and emission spectra (Figure S13) at room temperature for solid samples were recorded on Shimadzu RF-1501 spectrometer with 150W xenon lamp excitation source and 10 nm width of excitation and emission monochromator slits. Temperature dependence of photoluminescence emission spectra (400–720 nm) in temperature range of -150..+80ºC were measured using OceanOptics USB2000 fiber spectrometer incorporated into the originally designed setup (Figure S14). Emission spectra were collected with 2 sec intervals along with sample temperature change (cooling/heating rate 10 °/min).

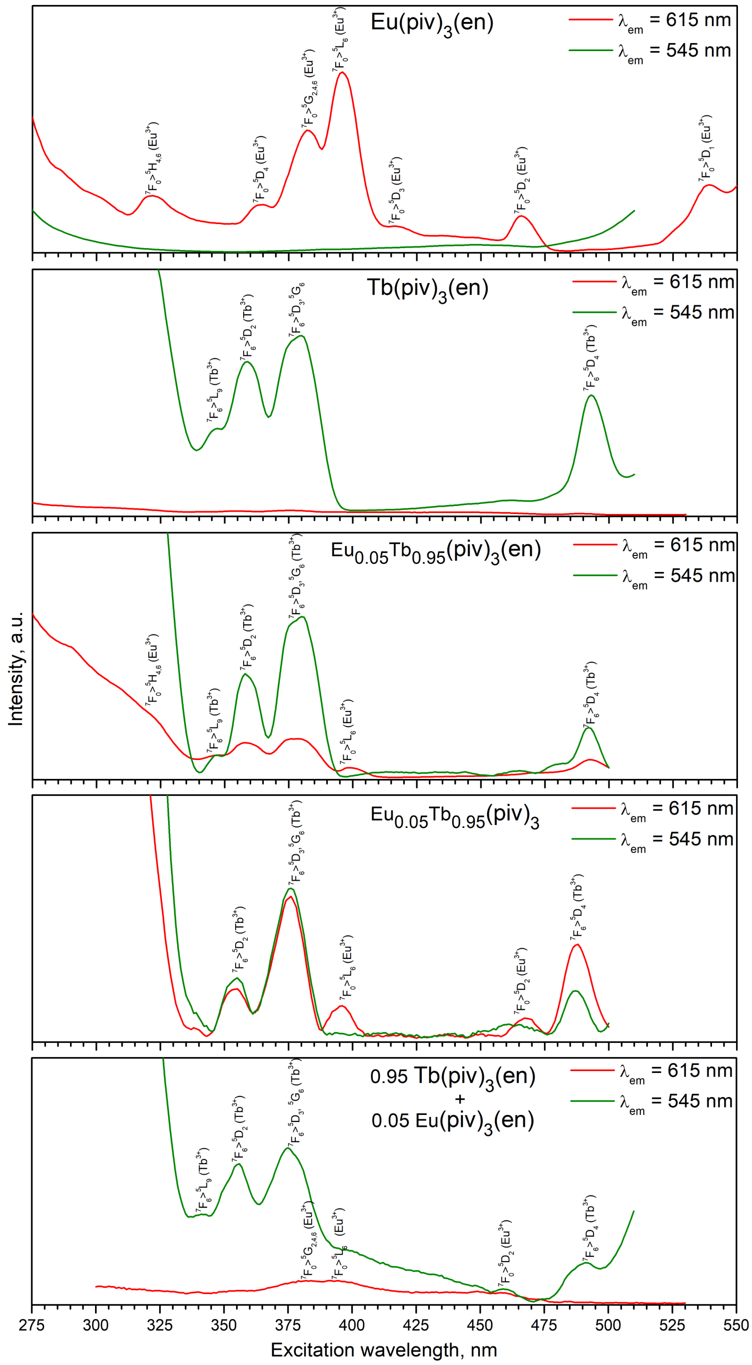

Figure S13. Photoluminescence excitation spectra of the Eu(piv)$_3$, Tb(piv)$_3$, Eu$_{0.05}$Tb$_{0.95}$(piv)$_3$, Eu$_{0.05}$Tb$_{0.95}$(piv)$_3$(en), and mechanical mixture Tb(piv)$_3$ and Eu(piv)$_3$ monitored at the emission wavelength of 545 nm (Tb^{3+}: $^5D_4 \rightarrow {}^7F_5$) and 615 nm ($Eu^{3+}$: $^5D_0 \rightarrow {}^7F_2$).

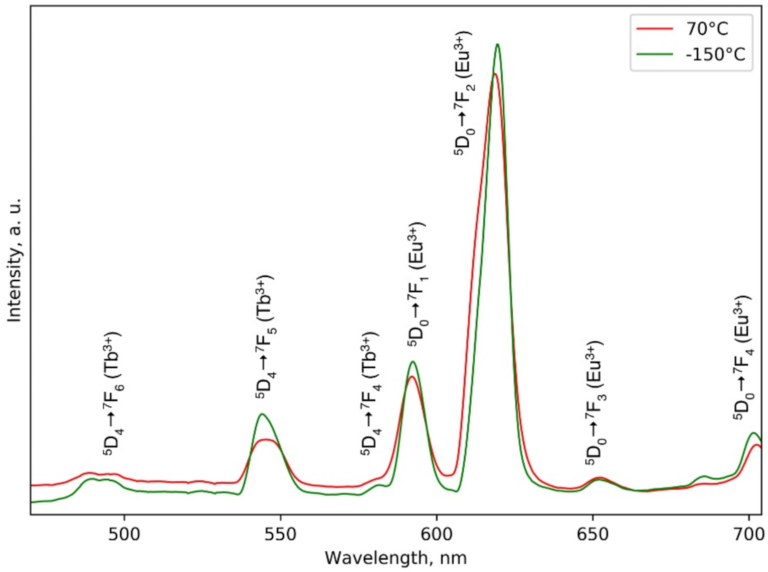

Figure S14. Photoluminescence emission spectra of the $Eu_{0.05}Tb_{0.95}(piv)_3$ at high and low temperatures. Excitation wavelength 365 nm.

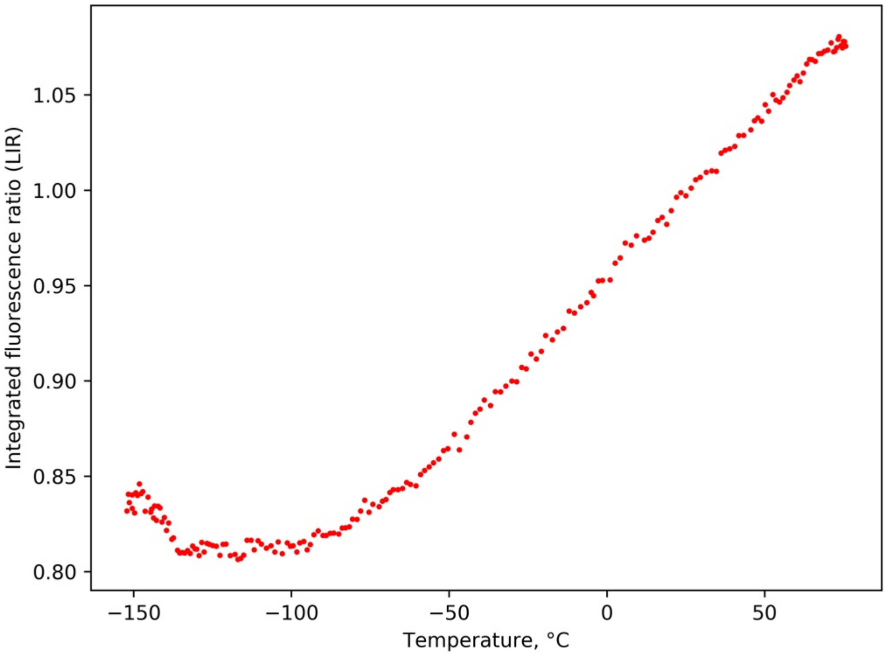

Figure S15. Temperature dependence of integrated luminescent intensity ratio of $^5D_0 \rightarrow ^7F_2$ transition of Eu^{3+} (615 nm) and $^5D_4 \rightarrow ^7F_5$ transition of Tb^{3+} (545 nm) in $Eu_{0.05}Tb_{0.95}(piv)_3(en)$. Excitation wavelength 365 nm.

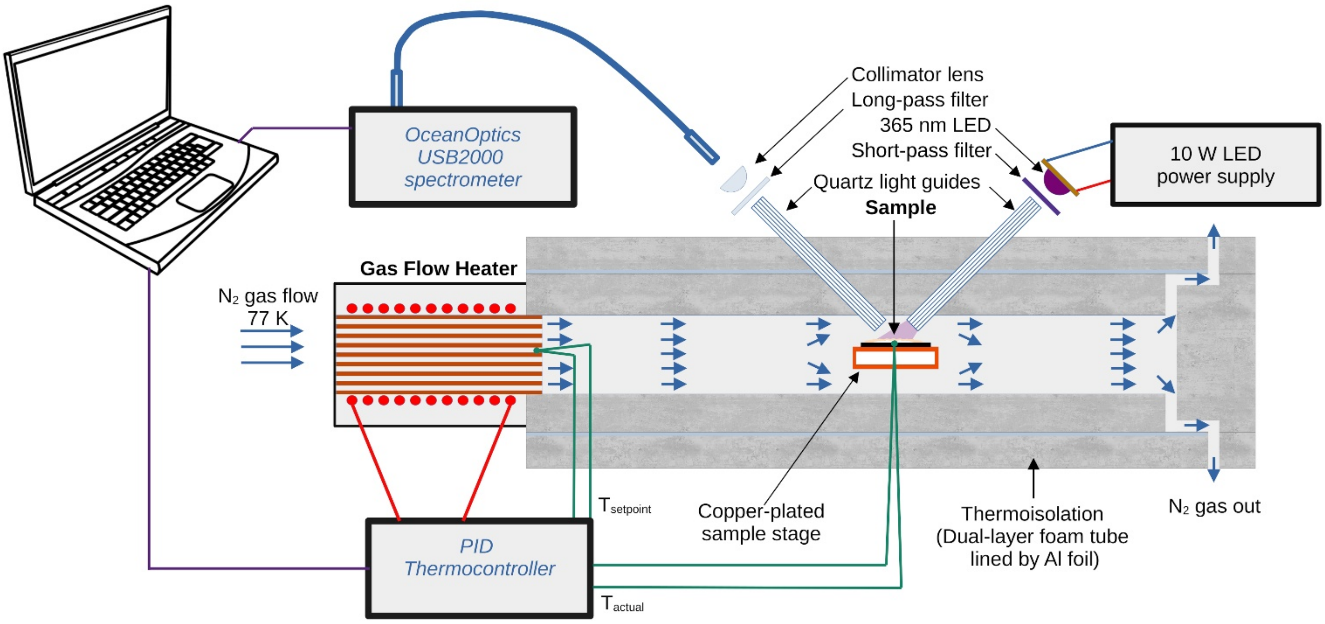

Figure S16. Scheme of experimental setup for temperature dependence of photoluminescence emission spectra measurements. Sample powder is fixed by carbon tape on copper-plated sample stage together with thin-wire K-type thermocouple (Tactual). The heating and cooling of sample stage occurs due to the nitrogen gas flow of a certain temperature (Tsetpoint). The temperature of gas flow from liquid N_2 boiler is regulated by Gas Flow Heater with PID thermocontroller attached directly to thermoisolation of optical chamber. Actual sample temperature and emission spectra in quazi real-time mode were stored on the PC for further processing and analysis.

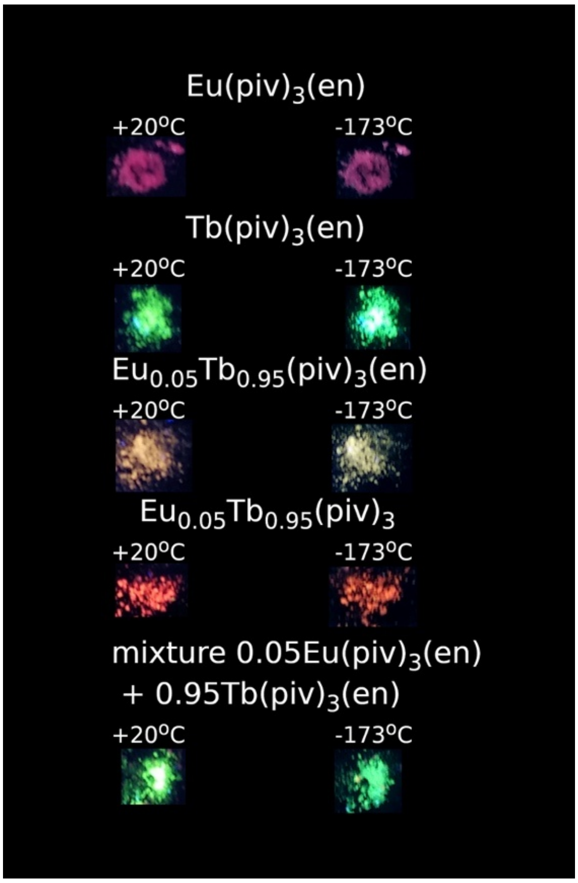

Figure S17. Color of the compounds under 365 nm LED illumination at high and low temperatures.

Table S5. CIE color coordinates of $Eu_{0.05}Tb_{0.95}(piv)_3$ and $Eu_{0.05}Tb_{0.95}(piv)_3(en)$ under 365 nm LED illumination at high and low temperatures.

Compound	T, °C	CIE x	CIE y
$Eu_{0.05}Tb_{0.95}(piv)_3$	−70	0.46	0.36
	70	0.48	0.34
$Eu_{0.05}Tb_{0.95}(piv)_3(en)$	−70	0.30	0.36
	70	0.37	0.40

References

[1] D. Casanova, M. Llunell, P. Alemany, S. Alvarez, *Chem. - A Eur. J.* **2005**, *11*, 1479–1494.

[2] S. L. J. Thomae, N. Prinz, T. Hartmann, M. Teck, S. Correll, M. Zobel, *Rev. Sci. Instrum.* **2019**, *90*.

[3] P. Juhás, T. Davis, C. L. Farrow, S. J. L. Billinge, *J. Appl. Crystallogr.* **2013**, *46*, 560–566.

[4] P. Juhás, C. L. Farrow, X. Yang, K. R. Knox, S. J. L. Billinge, *Acta Crystallogr. Sect. A Found. Adv.* **2015**, *71*, 562–568.
